# Simvastatin Enhances the Immune Response Against *Mycobacterium tuberculosis*

**DOI:** 10.3389/fmicb.2019.02097

**Published:** 2019-09-20

**Authors:** Paola Del Carmen Guerra-De-Blas, Miriam Bobadilla-Del-Valle, Isabel Sada-Ovalle, Iris Estrada-García, Pedro Torres-González, Alejandro López-Saavedra, Silvia Guzmán-Beltrán, Alfredo Ponce-de-León, José Sifuentes-Osornio

**Affiliations:** ^1^Laboratorio de Microbiología Clínica, Departamento de Infectología, Dirección de Medicina, Instituto Nacional de Ciencias Médicas y Nutrición Salvador Zubirán, Mexico City, Mexico; ^2^Laboratorio de Inmunología Integrativa, Instituto Nacional de Enfermedades Respiratorias “Ismael Cosío Villegas”, Mexico City, Mexico; ^3^Departamento de Inmunología, Escuela Nacional de Ciencias Biológicas, Instituto Politécnico Nacional, Mexico City, Mexico; ^4^Unidad Biomédica de Investigación en Cáncer, Instituto Nacional de Cancerología, Mexico City, Mexico

**Keywords:** simvastatin, tuberculosis, immune response, cytokines, autophagy, apoptosis, host directed therapy

## Abstract

Tuberculosis remains a serious threat worldwide. For this reason, it is necessary to identify agents that shorten the duration of treatment, strengthen the host immune system, and/or decrease the damage caused by the infection. Statins are drugs that reduce plasma cholesterol levels and have immunomodulatory, anti-inflammatory and antimicrobial effects. Although there is evidence that statins may contribute to the containment of *Mycobacterium tuberculosis* infection, their effects on peripheral blood mononuclear cells (PBMCs) involved in the immune response have not been previously described. Using PBMCs from 10 healthy subjects infected with *M. tuberculosis* H37Rv, we analyzed the effects of simvastatin on the treatment of the infections in an *in vitro* experimental model. Direct quantification of *M. tuberculosis* growth (in CFU/mL) was performed. Phenotypes and cell activation were assessed via multi-color flow cytometry. Culture supernatant cytokine levels were determined via cytokine bead arrays. The induction of apoptosis and autophagy was evaluated via flow cytometry and confocal microscopy. Simvastatin decreased the growth of *M. tuberculosis* in PBMCs, increased the proportion of NKT cells in culture, increased the expression of co-stimulatory molecules in monocytes, promoted the secretion of the cytokines IL-1β and IL-12p70, and activated apoptosis and autophagy in monocytes, resulting in a significant reduction in bacterial load. We also observed an increase in IL-10 production. We did not observe any direct antimycobacterial activity. This study provides new insight into the mechanism through which simvastatin reduces the mycobacterial load in infected PBMCs. These results demonstrate that simvastatin activates several immune mechanisms that favor the containment of *M. tuberculosis* infection, providing relevant evidence to consider statins as candidates for host-directed therapy. They also suggest that future studies are needed to define the roles of statin-induced anti-inflammatory mechanisms in tuberculosis treatment.

## Introduction

Currently, treatment for susceptible tuberculosis involves the administration of several drugs over a 6 month period, which can be extended for up to 18 months for multidrug-resistant tuberculosis (World Health Organization [WHO], 2018). The main problems in achieving optimal results during tuberculosis treatment are the length of treatment and the occurrence of adverse effects that favor poor compliance, treatment failure and resistance (Tang et al., 2018). In addition, tuberculosis is associated with various pulmonary complications such as fibrosis, bronchiectasis, and long-term chronic obstructive pulmonary disease, even when a cure is achieved (van Kampen et al., 2018). Therefore, it is necessary to develop new drugs that shorten the treatment duration, improve the immune response, or reduce lung damage caused by infection.

Recently, it has been proposed that drugs that modify the host immune response to infection rather than drugs directly acting against the pathogen may be more effective for treatment. This type of drug treatment is known as host-directed therapy. The use of drugs that reduce excessive inflammation, prevent tissue damage, or improve treatment efficacy has been proposed to eliminate infection by modulating the host immune response. Using host-directed therapies as an adjunct to standard anti-TB treatment could also reduce the infection duration and eventually lead to decreased relapse rates (Machelart et al., 2017). These types of drugs, including rapamycin, metformin, valproic acid, ibuprofen, and statins, have also been shown to affect the growth of mycobacteria in infected cells in *in vitro* studies (Schiebler et al., 2015; Zumla et al., 2015).

The primary effect of statins is a reduction in plasma cholesterol levels. Their mechanism of action is based on inhibition of the enzyme 3-hydroxy-3-methylglutaryl-coenzyme A (HMG-CoA) reductase, which catalyzes the conversion of HMG-CoA to mevalonic acid, a precursor of cholesterol (National Clinical Guideline Centre, 2014). In addition to these effects, various other immunomodulatory effects have been reported that have been associated with better results in the treatment of several infectious diseases (Thomas et al., 2015). Some of these effects involve modification of the expression levels of receptors involved in antigen presentation, immune cell activation, and the regulation of cytokine production (De Loecker and Preiser, 2012).

*In vitro* studies of *Mycobacterium tuberculosis* infection have shown that statins reduce the growth of *M. tuberculosis* (Parihar et al., 2014). In particular, simvastatin increased the bactericidal effects of first-line drugs that are often used to treat tuberculosis (i.e., isoniazid, rifampicin, and pyrazinamide) in an experimental BALB/c mice model both *in vitro* and *in vivo* (Guerra-De-Blas et al., 2018).

Although there is evidence that statins may contribute to the containment of *M. tuberculosis* infection, their effects on cells involved in the immune response have not been previously described. The investigation of such effects is important, as pleiotropic outcomes have been shown to affect the ability of the immune response to control infection. Therefore, the aim of this study was to evaluate the effect of simvastatin on peripheral blood mononuclear cells (PBMCs) in terms of phenotype expression, function and control of *M. tuberculosis* infection, tested with *M. tuberculosis* H37Rv using an *in vitro* experimental model.

## Materials and Methods

PBMCs were obtained from leukocyte concentrates from 10 healthy blood donors (7 men and 3 women) with a mean age of 30.9 years (±5.22 SD) who visited the blood bank in a third-level care institution in Mexico City. The inclusion criteria were age over 18 years, no history of using immunosuppressive drugs, no active tuberculosis infection, and no use of antimicrobial drugs during the last 7 days before the sample was taken. All donors signed written informed consent. The study was approved by the Institutional Review Board (Comité de Ética en Investigación, Instituto Nacional de Ciencias Médicas y Nutrición Salvador Zubirán) (REF. 1628).

### Mononuclear Cell Isolation

PBMCs were separated from a 20 mL sample of leukocyte concentrate via a Ficoll-Hypaque (Sigma-Aldrich, Oakville, Canada) density gradient (Boyum, 1976). The cells were counted using a TC20 automated cell counter (Bio-Rad, Hercules, CA, United States), and the cell viability was simultaneously evaluated using trypan blue staining. The cell pellet was resuspended at a concentration of 2 × 10^6^ cells/mL in RPMI-1640 medium (Gibco, Grand Island, NY, United States) supplemented with 10% fetal bovine serum and 2 mM glutamine without antibiotics.

### Preparation of *Mycobacterium tuberculosis* Cultures

*M. tuberculosis* H37Rv was cultured in Middlebrook 7H9 + OADC medium (Becton Dickinson, Sparks, MA, United States) supplemented with 0.2% glycerol and 0.05% Tween 80. The cultures were incubated at 37°C until they reached logarithmic phase (∼21 days). The bacterial mass was harvested via centrifugation, and 2 mL aliquots were prepared and stored at −70°C in cryovials containing Middlebrook medium.

For the immunofluorescence experiments, *M. tuberculosis* H37Rv was transformed with pCherry8 (provided by Addgene, Cambridge, MA, United States) via electroporation (Goude and Parish, 2009); this plasmid expresses the reporter fluorescent protein mCherry from the Psmyc promoter (Carroll et al., 2010). Next, the bacteria were cultured in Middlebrook 7H9 medium supplemented with OADC, 0.05% Tween-80 (Sigma-Aldrich), and hygromycin B (Roche Life Science, Indianapolis, IN, United States) at 37°C until they reached logarithmic phase (∼21 days). The bacterial mass was harvested via centrifugation, and 2 mL aliquots were prepared and stored at −70°C in cryovials containing Middlebrook medium.

### Preparation of the Simvastatin Stock Solution

Inactive simvastatin (lactone) (Sigma-Aldrich, Oakville, Canada) and either its active form or the beta-hydroxylated metabolite (β-hydroxy-acid or simvastatin acid) were used. Simvastatin or simvastatin acid concentrations ranging from 0.1 to 20 μM were tested. A 25 mM stock solution was prepared by dissolving 1.04 mg of simvastatin in a 30:70 (v/v) solution of dimethyl sulfoxide (DMSO) and absolute ethanol (EtOH). Subsequently, the stock solution was divided into two 500 μL aliquots. One aliquot was diluted in PBS (pH 7.4) to a concentration of 500 μM and sterilized via filtration through a 0.22 μm filter (Millipore, Carrigtwohill, Ireland); the other aliquot was activated via alkaline hydrolysis with 0.1 M NaOH at 50°C for 2 h, diluted in PBS (pH 7.4) to a concentration of 500 μM and then sterilized via filtration through a 0.22 μm filter (Rossi et al., 2010; Gordon et al., 2018).

### Treatment of *Mycobacterium tuberculosis* With Simvastatin and Direct Determination of the Number of Colony Forming Units (CFUs) via Plate Dilution

From the logarithmic phase culture of *M. tuberculosis* H37Rv, 2 mL was withdrawn and adjusted to a concentration equivalent to the 0.5 McFarland Standard (approximately 1.5 × 10^8^ CFU/ml) in Middlebrook 7H9 medium (Becton Dickinson) using a nephelometer (Biomerieux Vitek, Hach Co, Loveland, United States). Serial dilutions were made until a concentration of 1.5 × 10^4^ CFU/mL was obtained. In a 24-well plate, 1 mL of 1.5 × 10^4^ CFU/mL *M. tuberculosis* H37Rv was added in triplicate. Some of the samples were cultured in the presence of simvastatin or simvastatin acid, while others were cultured without treatment or with the vehicle (0.024% DMSO and 0.056% EtOH in PBS). Simvastatin and simvastatin acid were added at the following concentrations: 0.1, 0.5, 1, 2, 5, 10, 15, and 20 μM. The plates were incubated at 37°C in a 7.5% CO_2_ atmosphere for 96 h (Schaefer, 1957). To quantify the mycobacterial growth, serial dilutions were made. Ten microliter aliquots were obtained and inoculated onto Middlebrook 7H10 agar plates (Becton Dickinson) followed by incubation at 37°C in a 7.5% CO_2_ atmosphere. The plates were examined, and the CFU/mL counts were collected on days 7, 14, and 21.

### Infection of Mononuclear Cells With *Mycobacterium tuberculosis*

One vial of *M. tuberculosis* H37Rv logarithmic phase culture was thawed and centrifuged at 13,300 rpm for 10 min, and the supernatant was decanted. Subsequently, the bacterial pellet was resuspended in 1 mL of RPMI (Gibco, Carlsbad, CA, United States) supplemented with 10% fetal bovine serum, 1% non-essential amino acids (Gibco), 1% essential amino acids (Gibco), and 1% sodium pyruvate (Sigma) and then transferred to a 14 mL tube. To remove any bacterial lumps or aggregates, the suspension was passed through a 5 μm syringe filter (Millipore), and the single-cell count was obtained using a Petroff-Hausser chamber (Hausser Scientific, Horsham, PA, United States). The cells were infected at multiplicities of infection (MOIs) of 0.01, 0.1, and 1 (number of bacteria/cell).

### Infection of Mononuclear Cells With *Mycobacterium tuberculosis* and Treatment With Simvastatin

In each well of a 24-well plate, 1 mL of RPMI containing 2 × 10^6^ PBMC/mL was exposed to 1, 5, or 20 μM simvastatin or simvastatin acid. In addition, PBMCs treated with the vehicle (0.024% DMSO and 0.056% EtOH in PBS) and cells under basal conditions (cells cultured in RPMI) were also included. A total of 2 × 10^6^ PBMC/mL were infected at MOIs of 0.01, 0.1, and 1. The PBMCs were incubated in RPMI-1640 medium (Gibco) supplemented with 10% fetal bovine serum, 2 mM glutamine, 1% v/v non-essential amino acids, 1% v/v essential amino acids (Gibco), and 1% v/v sodium pyruvate (Sigma) at 37°C in a 5% CO_2_ atmosphere. Twenty-four hours after infection, the cell viability was evaluated via trypan blue staining using a TC-20 instrument (Bio-Rad). After 96 h, the PBMCs were lysed in sterile distilled water for 3 min. Subsequently, serial dilutions were made, and 10 μL aliquots were inoculated onto Middlebrook 7H10 agar plates (Becton Dickinson) to determine the CFU/mL.

### Immunophenotyping of Infected and Uninfected Mononuclear Cells Treated With Simvastatin

The PBMC concentration was adjusted to 2 × 10^6^ cells/mL, and 5 mL (1 × 10^7^ cells) of this suspension was seeded in RPMI medium in 25 mm^2^ cell culture flasks. The following conditions were used: (1) 1 μM simvastatin acid (SA1μM); (2) 20 μM simvastatin acid (SA20 μM); (3) 1 μM simvastatin (S1 μM); and (4) 20 μM simvastatin (S20 μM). The controls were cells exposed to the vehicle (0.024% DMSO and 0.056% EtOH in PBS) (No Tx) and cells exposed to phorbol myristate acetate (25 ng/mL) and ionomycin (1 μg/mL) (PMA + I). All flasks were incubated at 37°C in a 5% CO_2_ atmosphere. After 24 h, the phenotypes and activation status of the helper T cells (CD4+), cytotoxic T cells (CD8+), NKT cells (CD3+ Vα24- J18+), NK cells (CD3- CD16 CD56+), and monocytes (CD14+) were evaluated.

Other culture flasks were incubated under the previously described conditions for 24 h, and the cells were subsequently infected with *M. tuberculosis* H37Rv at an MOI of 0.1 (IFX SA1 μM; IFX SA20 μM; IFX S1 μM; IFX 20 μM; IFX (cells exposed to the vehicle and infected); IFX PMA + I). Twenty four hours post-infection, the phenotypes and activation status of the helper T cells (CD4+), cytotoxic T cells (CD8+), NKT cells (CD3+ Vα24- J18+), NK cells (CD3- CD16 CD56+), and monocytes (CD14+) were evaluated. The antibody panel was: anti-CD25-FITC, anti-CD69-PE, anti-CD14-PE, anti-CD3-PercP, anti-CD8-PeCy7, anti-CD4-APC, anti-CD16-APC Cy7, anti-CD56-APC Cy7, anti-CD80-PercP, anti-CD86-PeCy7, anti-CD16-APC Cy7, and anti-Vα24-J18-APC (Biolegend, San Diego, CA, United States). The FcγR receptors were blocked prior to staining with 10% human AB serum. The samples were measured using a FACSCanto II flow cytometer (BD Biosciences), and the data were analyzed using FlowJo software (Tree Star Inc., Ashland, OR, United States). The cut-off point was established using the fluorescence minus one (FMO) strategy. Single-cell selection was based on cell size and granularity (FSC vs. SSC). First, the CD3- and CD3+ cells were selected. From the CD3- cells, the CD16+ and CD56+ cells were selected to identify the NK cells. From the CD3+ cells, those expressing CD4 or CD8 were selected. In turn, the CD3+ CD4+ Vα24-Jα18+ cells and CD3+ CD8+ Vα24- Jα18+ cells were sub-selected (Supplementary Figure 1A).

To identify the monocytes, cells were again selected based on size and granularity. Subsequently, the CD14+ cells were selected, and from these, the CD14+ monocytes (classical) and CD14+ CD16+ monocytes (non-classical) were selected. The expression of co-stimulatory molecules (CD80 and C86) in both the classical and non-classical monocytes was also examined. The mean fluorescence intensity (MFI), an indicator of the receptor density per cell, was evaluated to determine the C80 and CD86 expression levels in the monocytes (Supplementary Figure 1B).

### Functional Analysis of the Effects of Simvastatin on Cytotoxic T Cells, NK Cells, and NKT Cells via Degranulation Kinetics

The degranulation capacity of CD8+ T, NKT, and NK cells was evaluated by examining the degranulation kinetics using flow cytometry as previously described (Wang et al., 2012). Briefly, the PBMC concentration was adjusted to 2 × 10^6^ cells/mL; from this suspension, 5 mL (1 × 10^7^ cells) was seeded in RPMI medium contained in 25 mm^2^ cell culture flasks that were incubated under the six previously described conditions. Three microliters of previously titrated anti-CD107a-PE antibody was added to each culture. After 1 h of incubation, 2 μM monensin was added (Biolegend). Aliquots containing 1 × 10^6^ cells were withdrawn after 2 and 5 h of incubation, and the cells were labeled with anti-perforin-FITC, anti-CD107a-PE, anti-CD3-PercP, anti-CD8-PeCy7, anti-CD4 APC, anti-CD16 APC Cy7, anti-CD56 APC Cy7, and anti-Vα24-J18 APC (Biolegend) antibodies. The degranulation capacity was also evaluated in cells cultured under the previously described conditions and infected with *M. tuberculosis* H37Rv at an MOI of 0.1. Three microliters of anti-CD107a-PE antibody was added to each culture. After 1 h of incubation, 2 μM monensin was added (Biolegend). Aliquots containing 1 × 10^6^ cells were withdrawn after 2 and 5 h of incubation, and the cells were labeled with anti-perforin-FITC, anti-CD107a-PE, anti-CD3-PercP, anti-CD8-PeCy7, anti-CD4 APC, anti-CD16 APC Cy7, anti-CD56 APC Cy7, and anti-Vα24-J18 APC (Biolegend) antibodies. All samples were measured in a FACSCanto II flow cytometer (BD Biosciences), and the data were analyzed using FlowJo software (Tree Star Inc., Ashland, OR, United States). The cut-off point was established using the FMO strategy.

### Effects of Simvastatin on Cytokine Production by *Mycobacterium tuberculosis*-Infected and Uninfected Mononuclear Cells

The cytokine levels were measured in supernatant from cultures of *M. tuberculosis* H37Rv-infected (MOI of 0.1) and uninfected cells treated as previously described. Twenty four hours post-treatment or post-infection, the supernatants were collected and stored in cryotubes at −70°C until analysis. To determine the cytokine levels, a Cytokine Bead Array (CBA) kit (Becton Dickinson, Biosciences) was used according to the manufacturer’s instructions. The levels of IFN-γ, TNFα, IL-1β, IL12p70, IL-17A, and IL-10 were quantified. The samples were measured using a FACSCanto II flow cytometer (BD Biosciences).

### Evaluation of the Effects of Simvastatin on Apoptosis and Autophagy in *Mycobacterium tuberculosis*-Infected Mononuclear Cells

Five milliliters of RPMI containing 1 × 10^7^ cells was seeded in 25 mm^2^ flasks and cultured under the following conditions (those which showed the greatest effects in the previous tests): (1) 20 μM simvastatin acid (SA 20 μM); (2) untreated cells exposed to the vehicle (0.024% DMSO and 0.056% EtOH in PBS); (3) cells infected at an MOI of 0.1; and (4) cells infected at an MOI of 0.1 and treated with 20 μM simvastatin acid. The cells were incubated at 37°C in a 5% CO_2_ atmosphere for 24 h. Subsequently, the induction of apoptosis and autophagy in these cells was evaluated.

To evaluate the induction of apoptosis, the CV-Caspase 3 and 7 detection kit (Enzo Life Sciences, NY, United States) was used. This kit contains CR(DEVD)2, a substrate that fluoresces after caspase 3 and 7 activation. PBMCs treated with 100 μM etoposide for 8 h were used as a positive control.

To measure autophagy, the CYTO-ID^®^ 2.0 autophagy detection kit (Enzo Life Sciences) was used. This kit stains autophagic compartments while ensuring minimal staining of lysosomes (Guo et al., 2015). PBMCs treated with 500 nM rapamycin for 8 h were used as a positive control.

To distinguish between the induction of autophagy in helper T cells, cytotoxic T cells, NKT cells, NK cells, and monocytes, the following monoclonal antibodies were used: anti-CD14 PE, anti-CD3 coupled to PercP, anti CD8-PeCy7, anti-CD4 APC, anti-CD16 APC-Cy7, anti-CD56 APC-Cy7, anti-CD16 APC-Cy7, and anti-Vα24-J18 APC (Biolegend). The FMO strategy was used to establish the cut-off points. For each cell subtype, the percentages of cells stained with CYTO-ID^®^ were compared between the different treatments (rapamycin; 20 μM simvastatin; infection with *M. tuberculosis* H37Rv; and infection with *M. tuberculosis* H37Rv and 20 μM simvastatin).

The same strategy [comparison of the percentages of cells stained with CR (DEVD)2] was used to evaluate the effects of simvastatin on the induction of apoptosis. The samples were measured using a FACSCanto II flow cytometer (BD Biosciences) (Supplementary Figure 2).

Additionally, microphotographs were obtained from an experiment in which 2 mL of PBMCs (2 × 10^6^ cells/mL) were seeded in RPMI medium in a 24-well plate and then subjected to the following conditions: (1) infection with the *M. tuberculosis* H37Rv-mCherry strain at an MOI of 1, and (2) infection with the *M. tuberculosis* H37Rv-mCherry strain at an MOI of 1 and treatment with 20 μM simvastatin acid. The cells were incubated at 37°C in a 5% CO_2_ atmosphere for 24 h. Subsequently, the cells were stained using the CYTO-ID^®^ 2.0 kit (Enzo Life Sciences) and with Hoechst 33342 (FABRICANTE) according to the manufacturer’s instructions. The cells were fixed in 4% paraformaldehyde. To adhere the cells to the coverslip, the PBMCs were centrifuged at 1800 rpm for 3 min in a Cytopro^®^ Cytocentrifuge Series 2 centrifuge (Puteaux, France). The cells were observed using confocal fluorescence microscopy with an LSM 710-DUOX inverted system (Carl Zeiss AG, Germany), and images were obtained using ZEN 2009 software (version 6.0, SP2; Carl Zeiss AG, Germany).

### Statistical Analysis

To compare two groups of non-parametric data, the Mann–Whitney *U*-test was used. For comparisons among four groups, the Kruskal–Wallis test was used. The data were analyzed using GraphPad Prism v6.0 software (La Jolla, CA, United States). A *p* ≤ 0.05 was considered to denote statistical significance. The results are expressed as the mean ± standard error.

## Results

### Direct Effects of Simvastatin on *Mycobacterium tuberculosis* Growth

**FIGURE 1:**
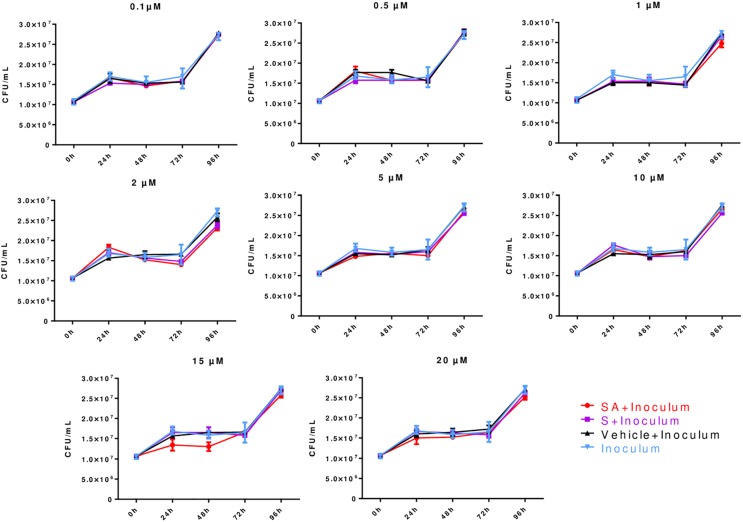
Direct effects of simvastatin on *Mycobacterium tuberculosis* growth expressed in colony forming units (CFUs). In a 24-well plate, 1.5 × 10^4^ CFU/mL *M. tuberculosis* H37Rv was added in triplicate and cultured in the presence of simvastatin acid (SA, red dots), simvastatin (S, purple squares), vehicle (0.024% DMSO and 0.056% EtOH in PBS, black triangles), and without any treatment/inoculum (blue triangles). The plates were incubated at 37°C in a 7.5% CO_2_ atmosphere for 24, 48, 72, or 96 h. Serial dilutions were made on Middlebrook 7H10 agar to determine the CFU/mL. Colonies were counted on days 7, 14, and 21. The simvastatin concentrations are indicated at the top of each graph, *n* = 5 (*p* > 0.05).

Different simvastatin concentrations (0.1–20 μM) were tested. The analysis of the growth kinetics showed that simvastatin, regardless of its activation state or concentration, had no direct bactericidal effects (*p* > 0.05; Figure 1).

### Effects of Simvastatin on *Mycobacterium tuberculosis* Growth in Infected Mononuclear Cells

**FIGURE 2:**
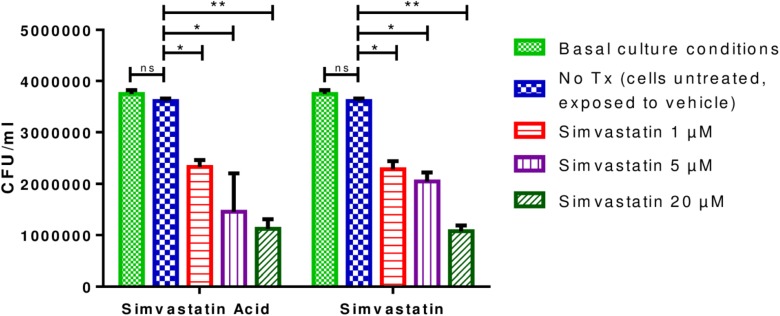
Effects of simvastatin on the growth of *Mycobacterium tuberculosis* in peripheral blood mononuclear cells. In a 24-well plate, 2 × 10^6^ PBMCs/mL were seeded and exposed to 1, 5, and 20 μM simvastatin and simvastatin acid. PBMCs treated with the vehicle (0.024% DMSO and 0.056% EtOH in PBS) and cells under basal conditions (cells cultured in RPMI) were included as controls. The cells were infected at an MOI of 0.1. The cells were incubated at 37°C in a 5% CO_2_ atmosphere. After 96 h, the cells were lysed, and serial dilutions were made. The serial dilutions were inoculated in triplicate onto Middlebrook 7H10 agar plates for CFU/mL determination, *n* = 10 (Kruskal–Wallis, ^∗^*p* < 0.05, ^∗∗^*p* < 0.01).

The optimal MOI for the infection of PBMCs resulted on 0.1 because an MOI of 1 induced cell death and a lower MOI induced very few phenotypic changes in the PBMCs (Supplementary Figure 3). It was observed that PBMCs that were infected and incubated for 96 h and treated with 1 μM simvastatin or 1 μM simvastatin acid, exhibited reduced bacterial loads compared to those of the control cells and/or those treated with the vehicle (reduction from 3.75 × 10^6^ to 6.88 × 10^5^ CFU/mL; *p* < 0.05). The decrease in bacterial load was even greater at a concentration of 20 μM (reduction from 1.92 × 10^7^ CFU/mL to 5.35 × 10^6^ CFU/mL; *p* < 0.001; Figure 2). The effects of the two forms of simvastatin were dose-dependent. Based on these results, it was decided that concentrations of 1 and 20 μM would be used in the subsequent experiments.

### Effects of Simvastatin on Phenotypes of Uninfected Mononuclear Cells

The effect of simvastatin on the cell viability of PBMCs was evaluated using trypan blue staining and the automated cell counter TC-20. No cytotoxic effect was observed, even using pharmacological concentrations (Supplementary Figure 4).

**FIGURE 3:**
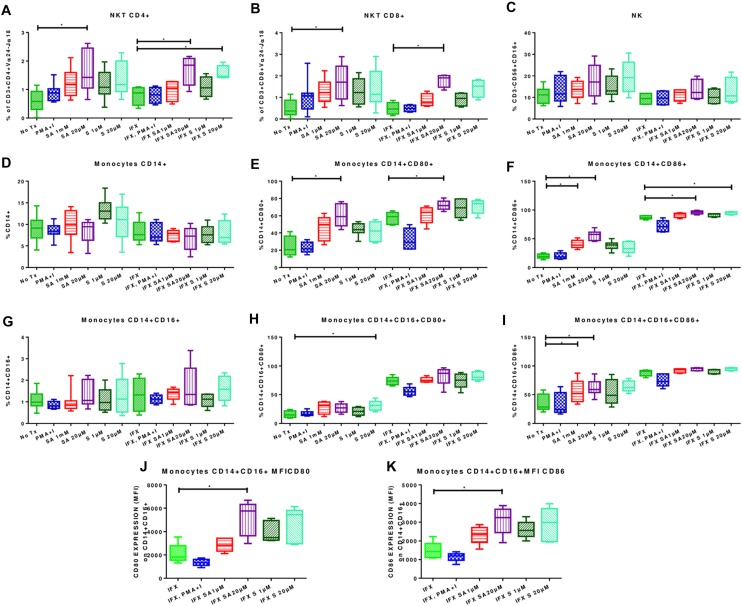
Effects of simvastatin on the phenotypes of infected and uninfected peripheral mononuclear cells. PBMCs were incubated under the following conditions: 1 μM simvastatin acid (SA1 μM), 20 μM simvastatin acid (SA20 μM), 1 μM simvastatin (S1 μM), and 20 μM simvastatin (S20 μM). The control cells were untreated cells exposed to vehicle (0.024% DMSO and 0.056% EtOH in PBS) (No Tx), or cells treated with phorbol myristate acetate (25 ng/mL) plus ionomycin (1 μg/mL). Other culture flasks were incubated under the previously described conditions for 24 h, and the cells were subsequently infected with *M. tuberculosis* H37Rv at an MOI of 0.1 (IFX SA1 μM; IFX SA20 μM; IFX S1 μM; IFX 20 M m; IFX (cells exposed to the vehicle and infected); IFX PMA + I). After 24 h, the phenotypes of the NKT cells **(A,B)**, NK cells **(C)**, and monocytes **(D,G)** were evaluated. The effects of simvastatin on the expression levels of CD80 **(E,H)** and CD86 **(F,I–K)** in monocytes are also shown, *n* = 10 (Kruskal–Wallis, ^∗^*p* < 0.05, ^∗^*p* < 0.01).

Treatment with 20 μM simvastatin increased the percentages of CD4+ NKT cells (1.63% ± 0.70 vs. 0.57% ± 0.3780; *p* < 0.05) and CD8+ NKT cells (1.69% ± 0.522 vs. 0.52% ± 0.38; *p* < 0.05) (Figures 3A,B; left side). Representative dot plots for the effect of simvastatin on NKTs are shown in Supplementary Figure 5. Simvastatin treatment did not affect the percentages of NK (Figure 3C). Treatment with simvastatin did not increase the percentage of classical and non-classical monocytes (Figures 3D,G; left side). However, in classical and non-classical monocytes, simvastatin treatment increased the expression levels of the co-stimulatory molecules CD80 (CD14+ CD80+, 60.28% ± 12.98 vs. 23.71% ± 11.44, p < 0.05; CD14+ CD16+ CD80+, 30.91% ± 8.33 vs. 15.69 ± 5.68; Figures 3E,H; left side) and CD86 (CD14+ CD86+, 55.64% ± 8.44 vs. 19.49% ± 3.9; CD14+ CD16+ CD86+, 64.45% ± 9.29 vs. 34.73% ± 14.52; Figures 3F,I; left side). Representative dot plots for the effect of simvastatin on the expression of CD80 and CD86 in monocytes are shown in Supplementary Figure 6.

Simvastatin treatment did not affect the percentages or activation status of CD4+ T and CD8+ T cells based on the measurement of the expression levels of CD25 and/or CD69 (Supplementary Figure 7).

### Effects of Simvastatin on Phenotypes of Mononuclear Cells Infected With *Mycobacterium tuberculosis*

Infected PBMCs exposed to 20 μM simvastatin showed an increase in the percentage of CD4+ NKT cells (1.7 ± 0.57 vs. 0.79 ± 0.33; *p* < 0.05) and CD8+ NKT cells (1.79 ± 0.31 vs. 0.48 ± 0.28; *p* < 0.05). Figures 3A,B; right side. Representative dot plots for the effect of simvastatin on NKTs are shown in Supplementary Figure 5.

Classical and non-classical monocytes showed no changes (Figures 3D,G; right side). However, the expression levels of CD80 and CD86 were increased in classical monocytes (CD14+ CD80+, 72.15% ± 5.6 vs. 57.31% ± 7.13; CD14+ CD86+, 95.92% ± 2.9 vs. 87.14% ± 3.14) (Figures 3E,F; right side). In non-classical monocytes, simvastatin had no effect on the expression levels of CD80 and CD86 (Figures 3H,I; right side). However, upon analyzing the CD80 and CD86 receptor densities, it was observed that simvastatin treatment increased the MFIs of CD80 (5142 ± 1482 vs. 2103 ± 379.6; *p* < 0.05) and CD86 (3108 ± 745.6 vs. 1476 ± 456.5; *p* < 0.05; Figures 3J,K) in these cells. Interestingly the simvastatin effect becomes more consistent when cells are infected. Representative dot plots for the effect of simvastatin on the expression of CD80 and CD86 in infected monocytes are shown in Supplementary Figure 6.

Infected lymphocytes showed increased expression levels of the activation markers CD25 and CD69, as expected, and exposure to simvastatin did not induce additional changes (Supplementary Figure 8).

### Effects of Simvastatin on Degranulation of CD8+ T Cells, NK Cells, and NKT Cells

Simvastatin treatment did not affect the degranulation of CD8+ T, NK, and NKT cells (Supplementary Figure 9). In addition, it was observed that when PBMCs were infected with *M. tuberculosis* (exposed and non-exposed to simvastatin) the CD107a expression increased and perforin expression decreased in CD8+ T cells, NK cells, and NKT cells after 5 h. This observation indicated that infection, *per se*, causes degranulation in these cells and that simvastatin did not induce additional changes.

### Effects of Simvastatin on Cytokine Production in Infected and Uninfected Mononuclear Cells

**FIGURE 4:**
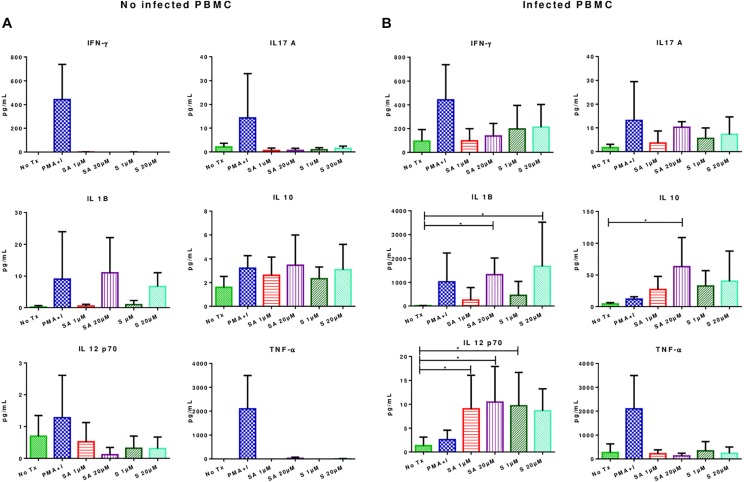
Effects of simvastatin on cytokine production in infected and uninfected peripheral mononuclear cells. **(A)** Effects of simvastatin on cytokine production in non-infected PBMCs. Cytokine production was measured in the supernatant of PBMCs incubated for 24 h with 1 μM simvastatin acid (SA1 μM), 20 μM simvastatin acid (SA20 μM), 1 μM simvastatin (S1 μM), and 20 μM simvastatin (S20 μM). The control cells were untreated cells exposed to vehicle (0.024% DMSO and 0.056% EtOH in PBS) (No Tx), or cells treated with phorbol myristate acetate (25 ng/mL) plus ionomycin (1 μg/mL). **(B)** Effects of simvastatin on cytokine production in infected PBMCs. The culture boxes were incubated in the previously described conditions for 24 h and subsequently, infected with *M. tuberculosis* H37Rv at an MOI of 0.1. At 24 h post-infection, the supernatant was collected, and cytokine production was measured, *n* = 10 (Kruskal–Wallis, ^∗^*p* < 0.05, ^∗^*p* < 0.01).

Simvastatin treatment did not result in significant changes in the secretion of IFN-γ, TNF-α, IL-1β, IL12-p70, IL-17A, and IL-10 in uninfected PBMCs (Figure 4A). However, in infected simvastatin-treated PBMCs, the levels of the pro-inflammatory cytokines IL-12-p70 (0.9 ± 1.79 pg/mL vs. 10.46 ± 7.48 pg/mL; *p* < 0.05) and IL-1β (14.7 ± 8.0 vs. 1676 ± 624.7; *p* < 0.05) were higher compared to their levels in untreated PBMCs. Simvastatin treatment also promoted IL-10 secretion in infected PBMCs (4.2 ± 1.911 vs. 63.2 ± 22.78; *p* < 0.05, see Figure 4B). No significant changes were observed in the production of IFN-γ, TNF-α, and IL-17A in infected PBMCs.

### Evaluation of the Effects of Simvastatin on Apoptosis and Autophagy in Infected and Uninfected Mononuclear Cells

In uninfected PBMCs treated with 20 μM simvastatin, an increase in the percentage of CR (DEVD)2-labeled PBMCs was observed compared with that in the untreated PBMCs, however, this difference was not statistically significant. Furthermore, an increase in the induction of apoptosis was observed in infected and treated PBMCs compared with infected non-treated PBMCs (37.3% ± 4.7 vs. 55.1% ± 5.8; *p* < 0.05).

**FIGURE 5:**
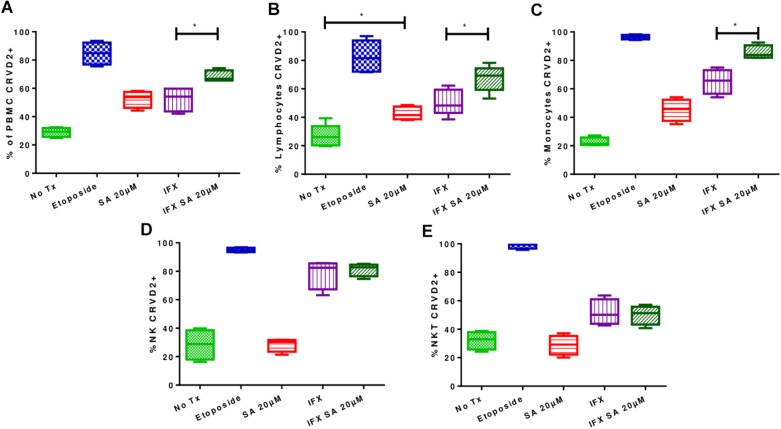
Effects of simvastatin on apoptosis in infected with *Mycobacterium tuberculosis* and uninfected peripheral mononuclear cells. The induction of apoptosis was evaluated in PBMCs treated for 24 h under the following conditions: 20 μM simvastatin acid (SA20 μM), untreated cells exposed to vehicle (0.024% DMSO and 0.056% EtOH in PBS) (No Tx), cells infected at an MOI of 0.1 (IFX), and cells infected at an MOI of 0.1 and treated with 20 μM simvastatin acid (IFX SA 20 μM). PBMCs treated with 100 μM etoposide for 8 h were used as a positive control for the evaluation of apoptosis. **(A)** Percentage of PBMCs positive for CR(DEVD)2; **(B)** percentage of lymphocytes positive for CR(DEVD)2; **(C)** percentage of monocytes positive for CR(DEVD)2; **(D)** percentage of NK cells positive for CR(DEVD)2; **(E)** percentage of NKT cells positive for CR(DEVD)2, *n* = 10 (Kruskal–Wallis, ^∗^*p* < 0.05, ^∗^*p* < 0.01).

Using flow cytometry, lymphocytes, monocytes and NK and NKT cells were examined to determine whether apoptosis was induced in all cell types or only in specific cell types. Figure 5 shows that simvastatin did not induce activation of the caspase pathway in NK and NKT cells, however, the percentage of CR (DEVD)2-stained lymphocytes was increased, indicating an increase in the induction of apoptosis (uninfected lymphocytes: 26.84% ± 7.86; uninfected and treated lymphocytes: 42.76% ± 4.56). In infected and treated PBMCs, the induction of apoptosis was shown to be greater in lymphocytes (50.6% ± 9.10 vs. 67.3% ± 9.2; *p* < 0.05) and monocytes (56.3% ± 13.2 vs. 83.6% ± 2.6; *p* < 0.05; Figure 5).

**FIGURE 6:**
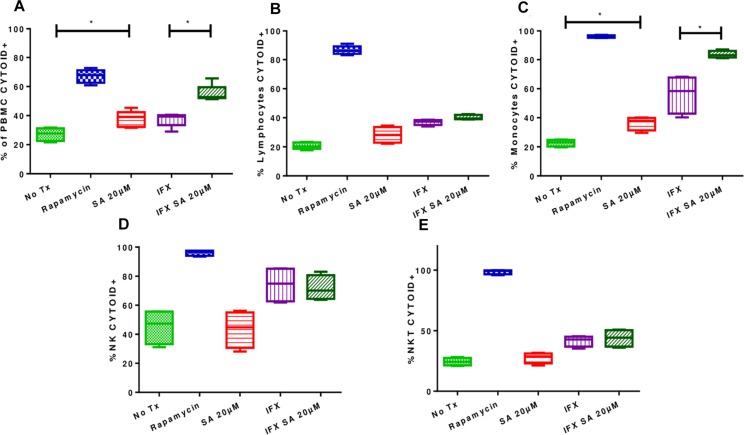
Effects of simvastatin on autophagy in infected and uninfected peripheral mononuclear cells. The induction of autophagy was evaluated in PBMCs treated for 24 h under the following conditions: 20 μM simvastatin acid (SA20 μM), untreated cells exposed to vehicle (0.024% DMSO and 0.056% EtOH in PBS) (No Tx), cells infected at an MOI of 0.1 (IFX), and cells infected at an MOI of 0.1 and treated with 20 μM simvastatin acid (IFX SA 20 μM). PBMCs treated with 500 nM rapamycin for 8 h were used as a positive control for the evaluation of autophagy. **(A)** Percentage of PBMCs positive for CYTO-ID; **(B)** percentage of lymphocytes positive for CYTO-ID; **(C)** percentage of monocytes positive for CYTO-ID; **(D)** percentage of NK cells positive for CYTO-ID; **(E)** percentage of NKT cells positive for CYTO-ID, *n* = 10 (Kruskal–Wallis, ^∗^*p* < 0.05, ^∗^*p* < 0.01).

**FIGURE 7:**
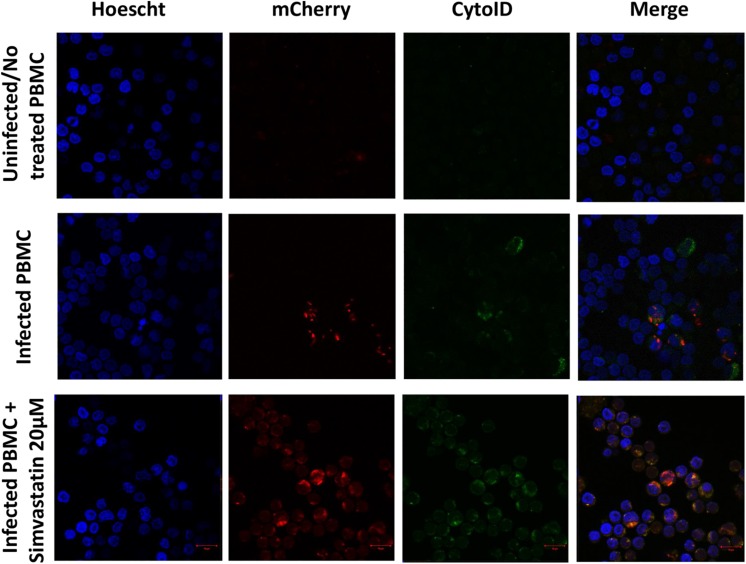
Induction of autophagy in peripheral blood mononuclear cells infected with *Mycobacterium tuberculosis* H37Rv-mCherry and treated with simvastatin. A total of 2 × 10^6^ PBMCs was seeded in a 24-well plate and cultured under the following conditions: (1) PBMCs infected at an MOI of 1 with *M. tuberculosis* strain H37Rv-mCherry, and (2) cells infected at an MOI of 1 with *M. tuberculosis* strain H37Rv-mCherry and treated with 20 μM simvastatin acid. Uninfected no treated cells were included as controls. The cells were incubated at 37°C in a 5% CO_2_ atmosphere for 24 h and then stained with Cyto-ID and Hoechst 33342 for detection of autophagosomes (green) and visualization of nuclei (blue), respectively. The stained cells were analyzed using confocal microscopy. (A) Intact mycobacteria were observed inside some infected PBMCs (red). The induction of autophagy was also observed independent of infection (green). (B) Sequestration of red stain within autophagosomes (green) and loss of the integrity of mycobacteria were observed in infected and treated PBMCs. Scale bar, 5 μm.

Uninfected and treated PBMCs exhibited an increase in the induction of autophagy (27.1% ± 4.5 vs. 37.6% ± 5.6; *p* < 0.05), and this effect was consistent in infected and treated PBMCs (37.36% ± 4.77 vs. 55.18% ± 5.8; *p* < 0.05; Figures 6A, 7). Microphotographs show intact mycobacteria inside in some infected PBMCs (red). The induction of autophagy was also observed independent of infection (green). In infected and treated PBMCs, sequestration of red stain within autophagosomes (green) and loss of *M. tuberculosis* integrity were observed (Figure 7).

Likewise, analyses of the results for each subpopulations (Figures 6B–E) showed that the induction of autophagy was greater in monocytes, both in uninfected and treated cells (22.65% ± 2.4 vs. 36.38% ± 4.72%) and in infected and treated cells (56.33% ± 13.27 vs. 83.63% ± 2.6%; *p* < 0.05; Figure 6C).

## Discussion

**FIGURE 8:**
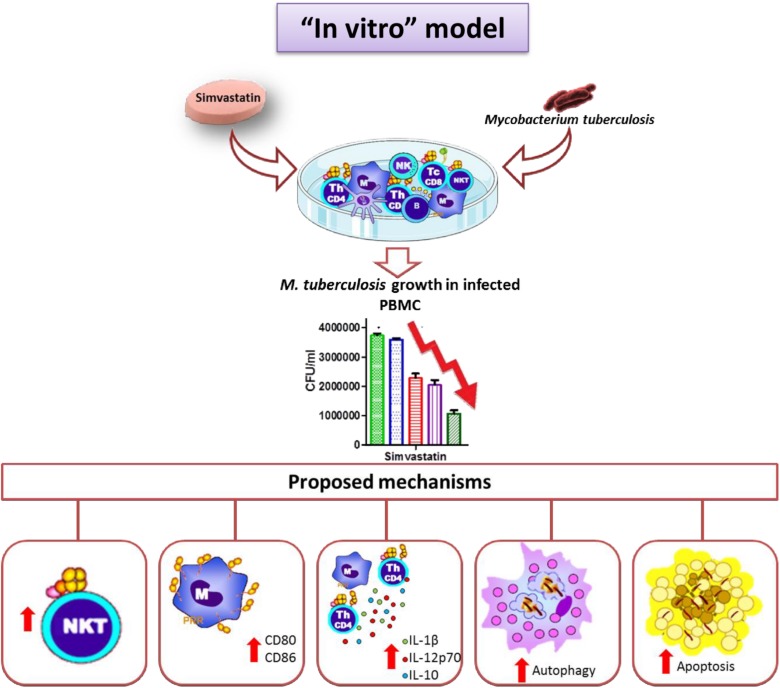
Simvastatin enhances the immune response against *Mycobacterium tuberculosis* in an *in vitro* model. Simvastatin decreased the growth of *M. tuberculosis* in PBMCs, increased the proportion of NKT cells in culture, increased the expression levels increased the proportion of NKT cells in culture, increased the expression of co-stimulatory molecules in monocytes, promoted the secretion of the cytokines IL-1β and IL-12p70, and activated apoptosis and autophagy in monocytes, resulting in a significant reduction in bacterial load. We also observed an increase in IL-10 production.

In this study, we described the effects of simvastatin on PBMCs in general and in terms of specific cell subtypes. We demonstrate that simvastatin and its metabolically active form (simvastatin acid) favors bacillus elimination via activation of several cellular mechanisms, including increased percentages of NKT cells within cultures, increased expression of co-stimulatory molecules on monocytes, higher secretion of the IL-1β and IL-12p70 cytokines, and activation of apoptosis and autophagy in monocytes. All of these effects, together, promoted improved *M. tuberculosis* elimination (Figure 8).

Simvastatin also favors IL-10 secretion and activated lymphocyte apoptosis, both of which are relevant phenomena that could inhibit adequate infection control, however, they could also mitigate the damage caused by excessive inflammation.

Regarding the possible bactericidal effects of simvastatin against *M. tuberculosis*, Skerry et al. (2014) tested concentrations up to 320 μM and did not observe any inhibitory effects. In contrast, Rens et al. (2016) observed an inhibitory effect at an MIC of 100 μg/mL (238.91 μM). In this study, we tested simvastatin concentrations ranging from 0.1 to 20 μM, which included the concentrations typically used in *in vitro* studies, and we did not detect any microbicidal effects. However, infected PBMCs treated with simvastatin exhibited a lower bacterial load.

Studies of the inhibitory effects of simvastatin on the growth of mycobacteria have been performed in macrophages (Parihar et al., 2014) and macrophage-like cell lines (THP-1, J774) (Lobato et al., 2014; Skerry et al., 2014). In this study, we evaluated the effects of simvastatin on the growth of *M. tuberculosis* H37Rv in PBMCs. The results showed simvastatin significantly reduced the growth of *M. tuberculosis* in infected PBMCs in a dose-dependent manner.

We also analyzed the effects of simvastatin on the cellular subtypes that were present in the experimental model. It was shown that simvastatin increased the percentages of CD4+ and CD8+ NKT cells. Nakou et al. (2012) reported that patients with hypercholesterolemia treated with simvastatin for at least 6 months showed increases in the number of peripheral blood NKT cells. On the other hand, several studies have reported a decrease in the number of NKT cells in subjects with active tuberculosis (Snyder-Cappione et al., 2007), and *in vitro* experiments have demonstrated the ability of NKT cells to kill *M. tuberculosis*-infected macrophages (Sada-Ovalle et al., 2008). Therefore, an increase in the percentages of CD4+ and CD8+ NKT cells is proposed to favor the protective immune response against mycobacteria.

We also observed that treatment with simvastatin at a pharmacological concentration (20 μM) increased the expression levels of the co-stimulatory molecules CD80 and CD86 in both infected and uninfected classical and non-classical monocytes. Arora et al. (2006) reported that simvastatin did not alter the expression levels of co-stimulatory molecules in dendritic cells; Atilla et al. observed that simvastatin treatment reduced the expression of CD83 and CD86 in dendritic cells derived from monocytes (Yilmaz et al., 2006). Gruenbacher et al. (2009) reported an increase in CD86 expression in simvastatin-treated CD14+ CD56+ cells. Burns et al. (2013) reported that in *S. aureus*-infected macrophages, simvastatin treatment did not alter the expression levels of the co-stimulatory CD80 and CD86 proteins. Under the experimental conditions used in this study, *M. tuberculosis*-infected PBMCs treated with simvastatin exhibited increased CD80 and CD86 expression in classical and non-classical monocytes. Consistently, Bhatt et al. reported that B7.1 and B7.2 (the murine homologs of CD80 and CD86) knockout mice are more susceptible to *M. tuberculosis* infection and that the B7 receptor signaling pathway is critical for long-term containment of infection within lung granulomas (Bhatt et al., 2009). In another study, cells recovered from induced sputum from patients with tuberculosis and controls were analyzed, and it was observed that cells from the infected patients had lower expression levels of co-stimulatory molecules (CD80 and CD86) (Flores-Batista et al., 2007). Decreased expression of these molecules, which are involved in antigen presentation and T-cell activation, in patients could lead to decreased T-cell recognition during *M. tuberculosis* infection. Therefore, a simvastatim-induced increase in the expression levels of these molecules, similar to that observed in this study, could favor T-cell recognition.

Cytokines are key for the initiation, maintenance, and modulation of the immune response against *M. tuberculosis*. In this study, we observed that simvastatin treatment increased IL- 1β and IL12p70 production in infected cells. The IL-1β cytokine is a key mediator of inflammation and plays an important role in host resistance against *M. tuberculosis* infection. Krishnan et al. reported that IL-1β knockout mice were more susceptible to *M. tuberculosis* infection and exhibited a greater bacterial load in the lungs and higher mortality at the beginning of the infection (Krishnan et al., 2013). Likewise, interleukin-12p70 (a heterodimer of the p35 and p40 subunits) induces bactericidal and cytolytic activity, increases the proliferation of macrophages, promotes IFN-γ production by NK cells, and plays a central role in polarization during the differentiation of T helper type 1 lymphocytes (Th1) during *M. tuberculosis* infection (Trinchieri, 2003; Khader et al., 2006). On the other hand, *M. tuberculosis* inhibits IL-12p40 production in macrophages to evade the immune system (Dao et al., 2008); therefore, increased IL-1β and IL-12p70 production is proposed as another mechanism by which simvastatin favors the inhibition of *M. tuberculosis* growth in infected cells.

Our results show that simvastatin-treated infected PBMCs produced more IL-10. It has been observed that IL-10 limits the Th1 responses against *M. tuberculosis* infection (Redford et al., 2011). However, a balanced immune response is very important in restricting the growth of mycobacteria without causing excessive tissue damage, as half of treated patients have permanent damage due to excessive inflammation caused by infection (Wallis and Hafner, 2015). Despite the increase in IL-10 production observed in our study, the CFU/mL counts demonstrated that simvastatin treatment favors the inhibition of *M. tuberculosis* growth in infected PBMCs. Thus, an increase in the production of this cytokine could inhibit excessive inflammation and reduce infection-induced damage.

Previous studies have reported that simvastatin has pro-apoptotic effects in cancerous cells and that it plays a role in the immune response against *M. tuberculosis* (Kamel et al., 2017). Consequently, we evaluated the effects of simvastatin on apoptosis in infected PBMCs. Our results showed that simvastatin induces apoptosis in PBMCs and does so primarily in macrophages and lymphocytes. Simvastatin-induced apoptosis in macrophages favors more effective elimination of the bacillus, while necrosis favors its dissemination (Fratazzi et al., 1997). However, apoptosis in lymphocytes could be unfavorable for the control of the infection (Cooper, 2009). Several studies have shown that the effectiveness of protective responses against *M. tuberculosis* infection depends mainly on its quality, as reflected in the ability of T cells to perform multiple functions, rather than on its magnitude (Orlando et al., 2018). On the other hand, Dutta et al. (2016) reported that statin therapy shortens the duration of anti-tuberculosis treatment in mice; therefore, it is necessary to study the impact of the induction of apoptosis in lymphocytes in more detail and to determine the appropriate time to start treatment with statins in this experimental model.

Parihar et al. (2014) reported that simvastatin promotes autophagy in *M. tuberculosis*-infected macrophages, however, its effect on other cell types and its possible involvement in the immune response against *M. tuberculosis* have not yet been analyzed. Likewise, our results show that the induction of autophagy occurs preferentially in monocytes, favoring the elimination of the bacillus via the formation of autophagosomes. The induction of autophagy by simvastatin seems to be critical, as this mechanism contributes to the elimination of intracellular microorganisms, such as *M. tuberculosis*; furthermore, autophagy controls inflammation and prevents tissue damage in animal models (Deretic, 2014; Olive and Sassetti, 2015).

With respect to the mechanism by which statins affect immune cells, it is known that when statins inhibit mevalonate synthesis, they also inhibit the production of other isoprenoid molecules, such as farnesyl pyrophosphate and geranylgeranyl pyrophosphate. These molecules serve as lipid labels for the post-translational modification of several proteins, including G protein gamma subunits and small GTP-binding proteins, such as Ras, Rho, Rab, Rac, Ral, or Rap (Oesterle et al., 2017). This last component (prenylation inhibition) has been proposed as the mechanism by which statins modulate the immune response. This study shows the effects of simvastatin on different cell types of the human immune system that enhance the elimination of *M. tuberculosis*, however, the effect is greater with the active metabolite, simvastatin acid. This outcome was expected because simvastatin is a lactone prodrug that inhibits HMG-CoA reductase after it is hydrolyzed into its active form *in vivo* (Schachter, 2005). Although the liver is the main organ that metabolizes statins, mononuclear cells express CYP3A and can also metabolize them (Temesvari et al., 2012).

This study has some limitations inherent to *in vitro* studies; for example, mononuclear cells from only 10 subjects (a small sample size) were included in the study, and the cells were obtained from healthy subjects and treated and infected outside of their “normal environment.” Therefore, it is necessary to conduct clinical studies to evaluate whether these beneficial effects are observed *in vivo* and to evaluate the therapeutic potential of statins as an adjuvant therapy for tuberculosis treatment. In this regard, Su et al. (2017) conducted a retrospective study that included 102,424 statin users and 202,718 subjects matched by age and sex. The two cohorts were monitored to detect the incidence of cases with active tuberculosis. They concluded that the use of statins was an independent protective factor for the development of tuberculosis (Su et al., 2017).

When considering the possible drug interactions of statins with current tuberculosis treatment, it is known that rifampicin decreases the simvastatin serum levels because it induces its metabolism, and the combination of isoniazid and simvastatin may increase the risk of myopathy and rhabdomyolysis because inhibits its metabolism (Saukkonen et al., 2006; Wiggins et al., 2016). Therefore, additional preclinical studies are needed to identify the optimal dosage with first-line drugs.

Regarding the drug interactions of antiretrovirals with statins, it has been reported that several antiretrovirals inhibit the metabolism of statins and, therefore, may increase the risk of muscle damage (rhabdomyolysis) (Chauvin et al., 2013). However, Bernal et al. (2017) reviewed clinical studies of statins in HIV-infected patients and concluded that statins used at the recommended therapeutic dose have a relatively low risk of adverse reactions and may even confer a favorable anti-inflammatory profile in this population. Regarding the use of statins as a possible adjuvant in anti-tuberculosis treatment in HIV patients, drug interactions represent a challenge; therefore, it is necessary to conduct carefully designed clinical studies that consider factors such as the type of statins used, their dose in combination therapies, and the duration of treatment, among others.

This study is the first to evaluate the effects of simvastatin on different subpopulations of PBMCs. These results are of great importance since the immune system involves complex interactions between diverse cell types in which the balance between the activation of pro-inflammatory and anti-inflammatory mechanisms is crucial to containing the infection while causing the least possible damage. Here, we have demonstrated that simvastatin exerted three different effects that favor the immune response against *M. tuberculosis* (increased the proportion of NKT cells in culture, increased the expression of co-stimulatory molecules in monocytes, and promoted the secretion of the cytokines IL-1β and IL-12p70) and activated two mechanisms that control cell death and promote *M. tuberculosis* killing (autophagy and apoptosis). These results provide new insights into the immune correlates that how simvastatin reduce mycobacterial load in PBMCs and endorses statins as potential candidates for host-directed therapy. This study not only provides an overview of the effects of simvastatin on PBMCs, it also suggests the possibility of future studies to define the roles of anti-inflammatory mechanisms induced by statins in the treatment of *M. tuberculosis* infections and to determine the most appropriate time to start treatment with these drugs. It will be necessary to conduct in vivo studies to determine whether statin treatment effectively prevents tissue damage resulting from *M. tuberculosis* infection.

## Data Availability

The datasets generated for this study are available on request to the corresponding author.

## Ethics Statement

PBMCs were obtained from leukocyte concentrates from 10 healthy blood donors (7 men and 3 women) with a mean age of 30.9 years (±5.22 SD) who visited the blood bank in a third-level care institution in Mexico City. The inclusion criteria were age over 18 years; no history of using immunosuppressive drugs; no active tuberculosis infection; and no antimicrobial drugs during the last 7 days before the sample was taken. All donors signed the written informed consent. The study was approved by the Institutional Review Board (Comité de Ética en Investigación, Instituto Nacional de Ciencias Médicas y Nutrición Salvador Zubirán) (REF. 1628).

## Author Contributions

PG-D-B, PT-G, and JS-O had the initial idea, which was developed into a project together with AP-D-L and MB-D-V. PG-D-B, AL-S, and SG-B performed the experiments. MB-D-V, AP-D-L, IS-O, and IE-G analyzed the data. AP-D-L, JS-O, and PG-D-B wrote the manuscript. PT-G, IE-G, AP-D-L, JS-O, and MB-D-V proofread the final version. All authors discussed and corrected the manuscript and approved it for publication.

## Conflict of Interest Statement

The authors declare that the research was conducted in the absence of any commercial or financial relationships that could be construed as a potential conflict of interest.

## References

[B1] Arora M. Chen L. Paglia M. Gallagher I. Allen J. E. Vyas Y. M. (2006). Simvastatin promotes Th2-type responses through the induction of the chitinase family member Ym1 in dendritic cells. *Proc. Natl. Acad. Sci. U.S.A.* 103 7777–7782. 10.1073/pnas.0508492103 16682645 PMC1472521

[B2] Bernal E. Marin I. Masia M. Gutierrez F. (2017). Statins in HIV- infected patients: potential beneficial effects and clinical use. *AIDS Rev.* 19 59–71. 28182617

[B3] Bhatt K. Uzelac A. Mathur S. McBride A. Potian J. Salgame P. (2009). B7 costimulation is critical for host control of chronic *Mycobacterium tuberculosis* infection. *J. Immunol.* 182 3793–3800. 10.4049/jimmunol.0802996 19265158

[B4] Boyum A. (1976). Isolation of lymphocytes, granulocytes and macrophages. *Scand. J. Immunol.* 5 9–15. 10.1111/j.1365-3083.1976.tb03851.x 1052391

[B5] Burns E. M. Smelser L. K. Then J. E. Stankiewicz T. E. Kushdilian M. McDowell S. A. (2013). Short term statin treatment improves survival and differentially regulates macrophage-mediated responses to *Staphylococcus aureus*. *Curr. Pharm. Biotechnol.* 14 233–241. 10.2174/1389201011314020014 23228241 PMC4467892

[B6] Carroll P. Schreuder L. J. Muwanguzi-Karugaba J. Wiles S. Robertson B. D. Ripoll J. (2010). Sensitive detection of gene expression in mycobacteria under replicating and non-replicating conditions using optimized far-red reporters. *PLoS One* 5:e9823. 10.1371/journal.pone.0009823 20352111 PMC2843721

[B7] Chauvin B. Drouot S. Barrail-Tran A. Taburet A. M. (2013). Drug-drug interactions between HMG-CoA reductase inhibitors (statins) and antiviral protease inhibitors. *Clin. Pharmacokinet* 52 815–831. 10.1007/s40262-013-0075-4 23703578

[B8] Cooper A. M. (2009). Cell-mediated immune responses in *Tuberculosis*. *Annu. Rev. Immunol.* 27 393–422. 10.1146/annurev.immunol.021908.132703 19302046 PMC4298253

[B9] Dao D. N. Sweeney K. Hsu T. Gurcha S. S. Nascimento I. P. Roshevsky D. (2008). Mycolic acid modification by the mmaA4 gene of M. *tuberculosis* modulates IL-12 production. *PLoS Pathog.* 4:e1000081. 10.1371/journal.ppat.1000081 18535659 PMC2390761

[B10] De Loecker I. Preiser J.-C. (2012). Statins in the critically ill. *Ann. Intensive Care* 2 19–19. 10.1186/2110-5820-2-19 22709377 PMC3488539

[B11] Deretic V. (2014). Autophagy in *Tuberculosis*. *Cold Spring Harb. Perspect. Med.* 4:a018481. 10.1101/cshperspect.a018481 25167980 PMC4208715

[B12] Dutta N. K. Bruiners N. Pinn M. L. Zimmerman M. D. Prideaux B. Dartois V. (2016). Statin adjunctive therapy shortens the duration of TB treatment in mice. *J. Antimicrob. Chemother.* 71 1570–1577. 10.1093/jac/dkw014 26903278 PMC5007636

[B13] Flores-Batista V. C. Boechat N. Lago P. M. Lazzarini L. C. Pessanha L. R. Almeida A. S. (2007). Low expression of antigen-presenting and costimulatory molecules by lung cells from *Tuberculosis* patients. *Braz. J. Med. Biol. Res.* 40 1671–1679. 10.1590/s0100-879x2006005000141 17713660

[B14] Fratazzi C. Arbeit R. D. Carini C. Remold H. G. (1997). Programmed cell death of Mycobacterium avium serovar 4-infected human macrophages prevents the mycobacteria from spreading and induces mycobacterial growth inhibition by freshly added, uninfected macrophages. *J. Immunol.* 158 4320–4327. 9126994

[B15] Gordon R. E. Zhang L. Peri S. Kuo Y. M. Du F. Egleston B. L. (2018). Statins synergize with hedgehog pathway inhibitors for treatment of Medulloblastoma. *Clin. Cancer Res.* 24 1375–1388. 10.1158/1078-0432.ccr-17-2923 29437795 PMC5856627

[B16] Goude R. Parish T. (2009). “Electroporation of Mycobacteria,” in *Mycobacteria Protocols*, Second Edn, eds Parish T. Brown A. C., (Totowa, NJ: Humana Press), 203–215. 10.1007/978-1-59745-207-6_13

[B17] Gruenbacher G. Gander H. Rahm A. Nussbaumer W. Romani N. Thurnher M. (2009). CD56+ human blood dendritic cells effectively promote TH1-type gammadelta T-cell responses. *Blood* 114 4422–4431. 10.1182/blood-2009-06-227256 19762486

[B18] Guerra-De-Blas P. D. C. Torres-González P. Bobadilla-Del-Valle M. Sada-Ovalle I. Ponce-de Léon A. Sifuentes-Osornio J. (2018). Potential effect of statins on *Mycobacterium tuberculosis* infection. *J. Immunol. Res.* 2018:14. 10.1155/2018/7617023 30581876 PMC6276473

[B19] Guo S. Liang Y. Murphy S. F. Huang A. Shen H. Kelly D. F. (2015). A rapid and high content assay that measures cyto-ID-stained autophagic compartments and estimates autophagy flux with potential clinical applications. *Autophagy* 11 560–572. 10.1080/15548627.2015.1017181 25714620 PMC4502761

[B20] Kamel W. A. Sugihara E. Nobusue H. Yamaguchi-Iwai S. Onishi N. Maki K. (2017). Simvastatin-induced apoptosis in osteosarcoma cells: a key role of RhoA-AMPK/p38 MAPK signaling in antitumor activity. *Mol. Cancer Ther.* 16 182–192. 10.1158/1535-7163.mct-16-0499 27799356

[B21] Khader S. A. Partida-Sanchez S. Bell G. Jelley-Gibbs D. M. Swain S. Pearl J. E. (2006). Interleukin 12p40 is required for dendritic cell migration and T cell priming after *Mycobacterium tuberculosis* infection. *J. Exp. Med.* 203 1805–1815. 10.1084/jem.20052545 16818672 PMC2118335

[B22] Krishnan N. Robertson B. D. Thwaites G. (2013). Pathways of IL-1beta secretion by macrophages infected with clinical *Mycobacterium tuberculosis* strains. *Tuberculosis* 93 538–547. 10.1016/j.tube.2013.05.002 23849220 PMC3759846

[B23] Lobato L. S. Rosa P. S. Ferreira Jda S. Neumann Ada S. da Silva M. G. do Nascimento D. C. (2014). Statins increase rifampin mycobactericidal effect. *Antimicrob. Agents Chemother.* 58 5766–5774. 10.1128/aac.01826-13 25049257 PMC4187984

[B24] Machelart A. Song O. R. Hoffmann E. Brodin P. (2017). Host-directed therapies offer novel opportunities for the fight against *Tuberculosis*. *Drug Discov. Today* 22 1250–1257. 10.1016/j.drudis.2017.05.005 28533187

[B25] Nakou E. Babageorgakas P. Bouchliou I. Tziakas D. N. Miltiades P. Spanoudakis E. (2012). Statin-induced immunomodulation alters peripheral invariant natural killer T-cell prevalence in hyperlipidemic patients. *Cardiovasc. Drugs Ther.* 26 293–299. 10.1007/s10557-012-6387-z 22441892

[B26] National Clinical Guideline Centre (2014). *Lipid Modification: Cardiovascular Risk Assessment, and the Modification of Blood Lipids for the Primary, and Secondary Prevention of Cardiovascular Disease.* London: National Institute for Health and Care Excellence. 25340243

[B27] Oesterle A. Laufs U. Liao J. K. (2017). Pleiotropic effects of statins on the cardiovascular system. *Circul. Res.* 120 229–243. 10.1161/circresaha.116.308537 28057795 PMC5467317

[B28] Olive A. J. Sassetti C. M. (2015). New TB treatments hiding in plain sight. *EMBO Mol. Med.* 7 125–126. 10.15252/emmm.201404815 25535253 PMC4328643

[B29] Orlando V. La Manna M. P. Goletti D. Palmieri F. Lo Presti E. Joosten S. A. (2018). Human CD4 T-cells with a naive phenotype produce multiple cytokines during *Mycobacterium tuberculosis* infection and correlate with active disease. *Front. Immunol.* 9:1119. 10.3389/fimmu.2018.01119 29875774 PMC5974168

[B30] Parihar S. P. Guler R. Khutlang R. Lang D. M. Hurdayal R. Mhlanga M. M. (2014). Statin therapy reduces the *Mycobacterium tuberculosis* burden in human macrophages and in mice by enhancing autophagy and phagosome maturation. *J. Infect. Dis.* 209 754–763. 10.1093/infdis/jit550 24133190

[B31] Redford P. S. Murray P. J. O’Garra A. (2011). The role of IL-10 in immune regulation during *M. tuberculosis* infection. *Mucosal. Immunol.* 4 261–270. 10.1038/mi.2011.7 21451501

[B32] Rens C. Laval F. Daffe M. Denis O. Frita R. Baulard A. (2016). Effects of lipid-lowering drugs on vancomycin susceptibility of mycobacteria. *Antimicrob. Agents Chemother.* 60 6193–6199. 10.1128/aac.00872-16 27503643 PMC5038262

[B33] Rossi J. Rouleau L. Emmott A. Tardif J. C. Leask R. L. (2010). Laminar shear stress prevents simvastatin-induced adhesion molecule expression in cytokine activated endothelial cells. *Eur. J. Pharmacol.* 649 268–276. 10.1016/j.ejphar.2010.09.016 20863785

[B34] Sada-Ovalle I. Chiba A. Gonzales A. Brenner M. B. Behar S. M. (2008). Innate invariant NKT cells recognize *Mycobacterium tuberculosis*-infected macrophages, produce interferon-gamma, and kill intracellular bacteria. *PLoS Pathog.* 4:e1000239. 10.1371/journal.ppat.1000239 19079582 PMC2588496

[B35] Saukkonen J. J. Cohn D. L. Jasmer R. M. Schenker S. Jereb J. A. Nolan C. M. (2006). An official ATS statement: hepatotoxicity of antituberculosis therapy. *Am. J. Respir. Crit. Care Med.* 174 935–952. 10.1164/rccm.200510-1666st 17021358

[B36] Schachter M. (2005). Chemical, pharmacokinetic and pharmacodynamic properties of statins: an update. *Fundam. Clin. Pharmacol.* 19 117–125. 10.1111/j.1472-8206.2004.00299.x 15660968

[B37] Schaefer W. B. (1957). Studies on the inhibiting effect of carbon dioxide on the growth of two mutant strains of *Mycobacterium tuberculosis*. *J. Bacteriol.* 73 52–55. 13405860 10.1128/jb.73.1.52-55.1957 PMC289744

[B38] Schiebler M. Brown K. Hegyi K. Newton S. M. Renna M. Hepburn L. (2015). Functional drug screening reveals anticonvulsants as enhancers of mTOR-independent autophagic killing of *Mycobacterium tuberculosis* through inositol depletion. *EMBO Mol. Med.* 7 127–139. 10.15252/emmm.201404137 25535254 PMC4328644

[B39] Skerry C. Pinn M. L. Bruiners N. Pine R. Gennaro M. L. Karakousis P. C. (2014). Simvastatin increases the *in vivo* activity of the first-line *Tuberculosis* regimen. *J. Antimicrob. Chemother.* 69 2453–2457. 10.1093/jac/dku166 24855121 PMC4184365

[B40] Snyder-Cappione J. E. Nixon D. F. Loo C. P. Chapman J. M. Meiklejohn D. A. Melo F. F. (2007). Individuals with pulmonary tuberculosis have lower levels of circulating CD1d-restricted NKT Cells. *J. Infect. Dis.* 195 1361–1364. 10.1086/513567 17397008

[B41] Su V. Y. Su W. J. Yen Y. F. Pan S. W. Chuang P. H. Feng J. Y. (2017). Statin use is associated with a lower risk of TB. *Chest* 152 598–606. 10.1016/j.chest.2017.04.170 28479115

[B42] Tang Z. Q. Jiang R. H. Xu H. B. (2018). Effectiveness of pharmaceutical care on treatment outcomes for patients with first-time pulmonary *Tuberculosis* in China. *J. Clin. Pharm. Ther*. 43 888–894. 10.1111/jcpt.12746 30003561

[B43] Temesvari M. Kobori L. Paulik J. Sárváry E. Belič A. Monostory K. (2012). Estimation of drug-metabolizing capacity by CYP-genotyping and CYP-expression. *J. Pharmacol. Exp. Ther.* 370:jet.111.189597. 10.1124/jpet.111.189597 22262920

[B44] Thomas G. Hraiech S. Loundou A. Truwit J. Kruger P. McAuley D. F. (2015). Statin therapy in critically-ill patients with severe sepsis: a review and meta-analysis of randomized clinical trials. *Minerva Anestesiol.* 81 921–930. 25690048

[B45] Trinchieri G. (2003). Interleukin-12 and the regulation of innate resistance and adaptive immunity. *Nat. Rev. Immunol.* 3 133–146. 10.1038/nri1001 12563297

[B46] van Kampen S. C. Wanner A. Edwards M. Harries A. D. Kirenga B. J. Chakaya J. (2018). International research and guidelines on post-tuberculosis chronic lung disorders: a systematic scoping review. *BMJ Glob. Health* 3:e000745. 10.1136/bmjgh-2018-000745 30057796 PMC6058174

[B47] Wallis R. S. Hafner R. (2015). Advancing host-directed therapy for *Tuberculosis*. *Nat. Rev. Immunol.* 15 255–263. 10.1038/nri3813 25765201

[B48] Wang J. Liu Z. Jiang L. P. An Y. F. Zhao X. D. (2012). [Screening for cytotoxic defects with flow cytometric detection of CD107alpha on natural killer cells and cytotoxic lymphocyte cells]. *Zhonghua Er Ke Za Zhi* 50 386–391. 22883044

[B49] Wiggins B. S. Saseen J. J. Page R. L. II Reed B. N. Sneed K. Kostis J. B. (2016). Recommendations for management of clinically significant drug-drug interactions with statins and select agents used in patients with cardiovascular disease: a scientific statement from the American heart association. *Circulation* 134:e468–e495. 27754879 10.1161/CIR.0000000000000456

[B50] World Health Organization [WHO] (2018). *Global Tuberculosis Report, Geneva, Licence: CC BY-NCSA3.0 IGO.* Geneva: WHO.

[B51] Yilmaz A. Reiss C. Weng A. Cicha I. Stumpf C. Steinkasserer A. (2006). Differential effects of statins on relevant functions of human monocyte-derived dendritic cells. *J. Leukocyte Biol.* 79 529–538. 10.1189/jlb.0205064 16387846

[B52] Zumla A. Maeurer M. Zumla A. Chakaya J. Hoelscher M. Ntoumi F. (2015). Host-directed therapies for tackling multi-drug resistant *Tuberculosis*: learning from the pasteur-bechamp debates. *Clin. Infect. Dis.* 61 1432–1438. 10.1093/cid/civ631 26219693

