# Supplementary material for: Simvastatin Enhances the Immune Response Against Mycobacterium tuberculosis
Source: Front Microbiol. 2019 Sep 20;10:2097. doi: 10.3389/fmicb.2019.02097 (PMC6764081; doi:10.3389/fmicb.2019.02097)
Supplement: Supplementary file 1 [file Data_Sheet_1.PDF]

Supplementary Figure 1

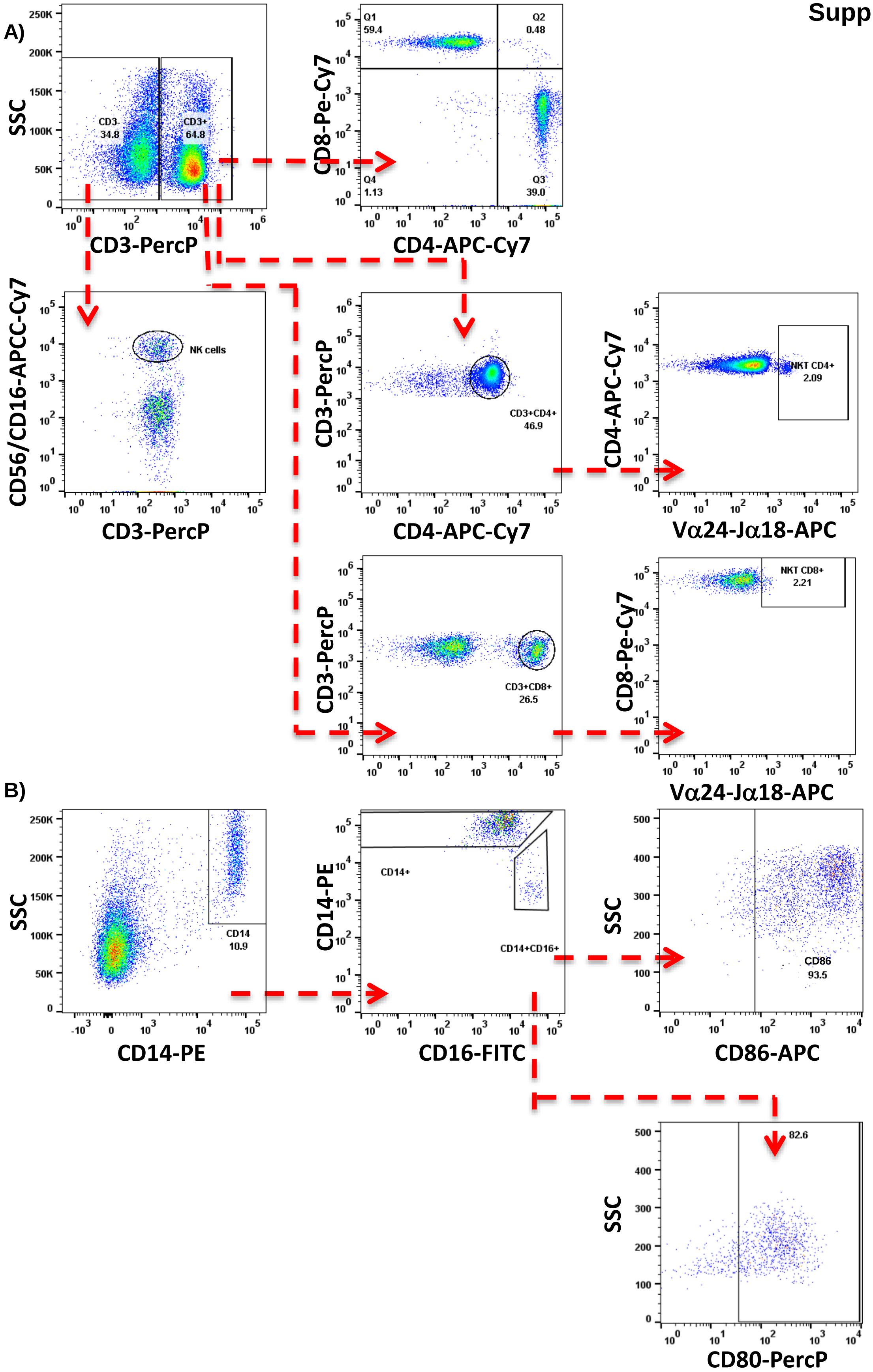

Supplementary Figure 1. Polychromatic flow cytometry gating tree. The cut-offs used for background fluorescence were based on isotype-matched Ig negative controls and the FMO (fluorescence minus one) strategy. A. Selection of CD4+ T cells, CD8+ T cells, NK cells, NKT CD4+ and NKT CD8+ lymphocytes. B. Selection of CD14+ (classical) and CD14+ CD16+ (non-classical) monocytes. Histograms show the selection of CD80 and CD86.

A)

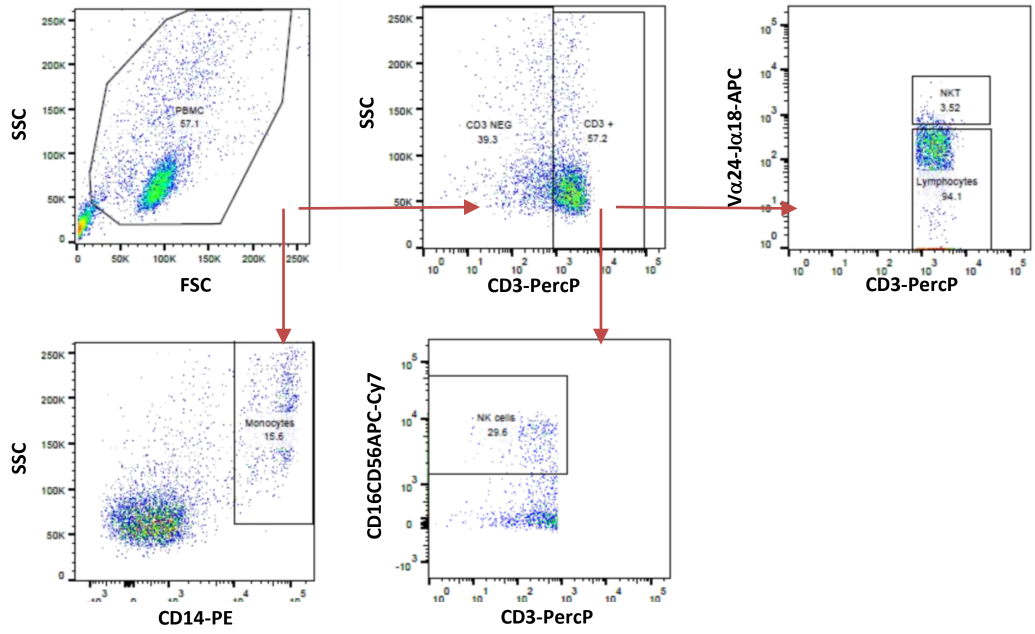

B)

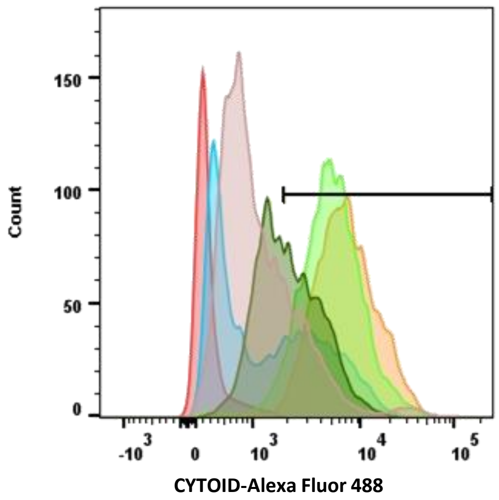

Colour	Condition	% CYTOID+
	SM	0%
	No TX	20.2%
	SA 20 µM	39.4%
	IFX	51.4%
	IFX SA 20 µM	84.7%
	Rapamycin 500 nM	97.5%

Supplementary Figure 2. Polychromatic flow cytometry gating tree used for detection of autophagy. The cut-offs used for background fluorescence were based the FMO (fluorescence minus one) strategy. The induction of autophagy was evaluated in PBMCs treated for 24 hours in the following conditions: 20 µM simvastatin acid (SA20µM), cells not treated with simvastatin (No Tx), cells to which only the vehicle was added (0.024% DMSO and 0.056% EtOH in PBS), cells infected at an MOI of 0.1 (IFX), and cells infected at an MOI of 0.1 and treated with simvastatin acid (IFX SA 20 µM). As a positive control for the evaluation of autophagy, PBMCs treated with 500 nM rapamycin for 8 hours were used. As a negative control, unstained PBMCs also were used (SM). A) Selection of TCD4+, TCD8+, NK, NK and NKT cells. B) Selection of cells positive for CYTO-ID.

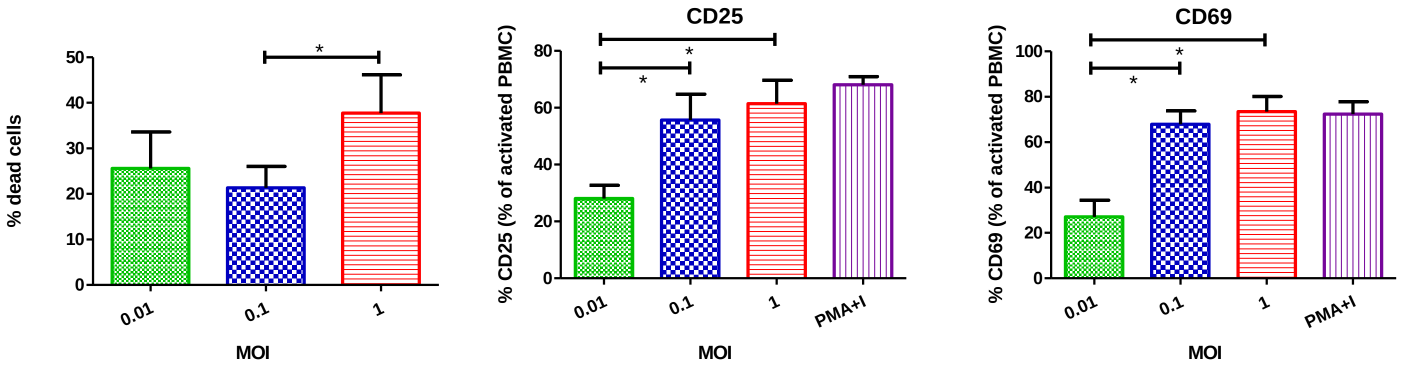

Supplementary Figure 3. Cell viability and activation of PBMCs infected with *Mycobacterium tuberculosis*. In a 24-well plate, $2x10^6$ PBMCs/mL were seeded and infected at diferent MOI of 0.01, 0.1 and 1. Cells were incubated at 37 °C in a 5% CO_2 atmosphere. At 24 hours post-infection, cell viability was evaluated using trypan blue staining. The percentages of PBMCs expressing the activation markers CD25 and CD69 were evaluated using flow cytometry, n=5. (Kruskal-Wallis, *$p < 0.05$ *$p < 0.01$).

Supplementary Figure 4

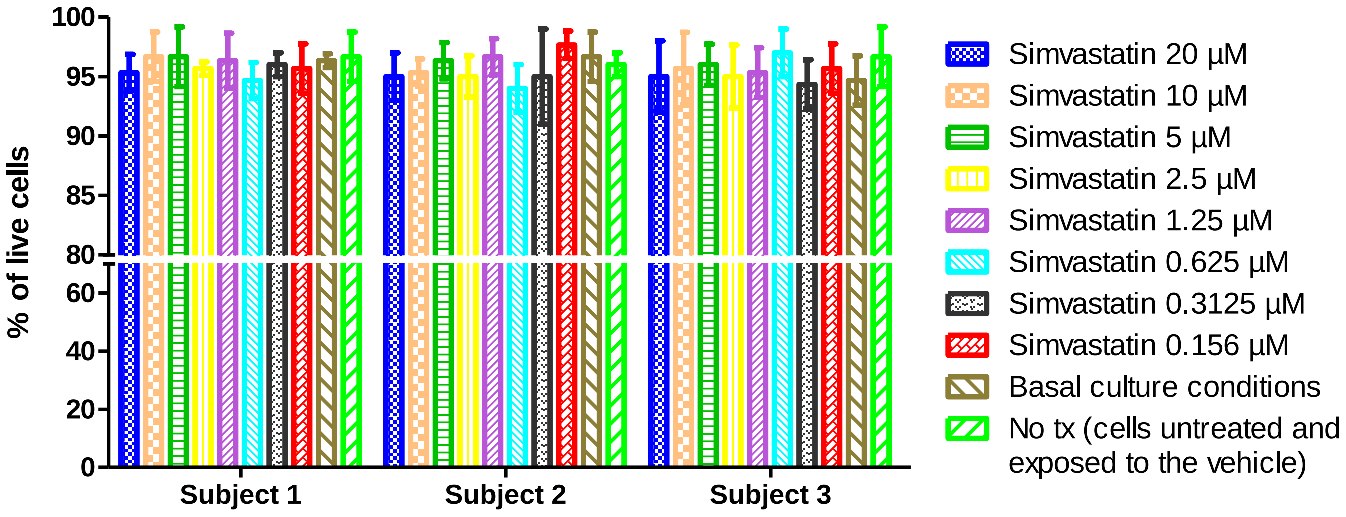

Supplementary Figure 4. Effect of simvastatin on the cell viability of PBMCs. In a 24-well plate, $1x10^6$ PBMCs/mL were seeded in triplicate and cultured in the presence of simvastatin at different concentrations). Cells untreated and exposed to the vehicle (0.024% DMSO and 0.056% EtOH in PBS, No Tx), and cells in basal culture conditions (supplemented RPMI) were included as controls. The plates were incubated at 37 °C in a 7.5% CO_2 atmosphere 96 hours. Then cell viability was evaluated using trypan blue staining and the automated cell counter TC-20. n=3. (Kruskal-Wallis, $p > 0.05$). No cytotoxic effect was observed, even using pharmacological concentrations.

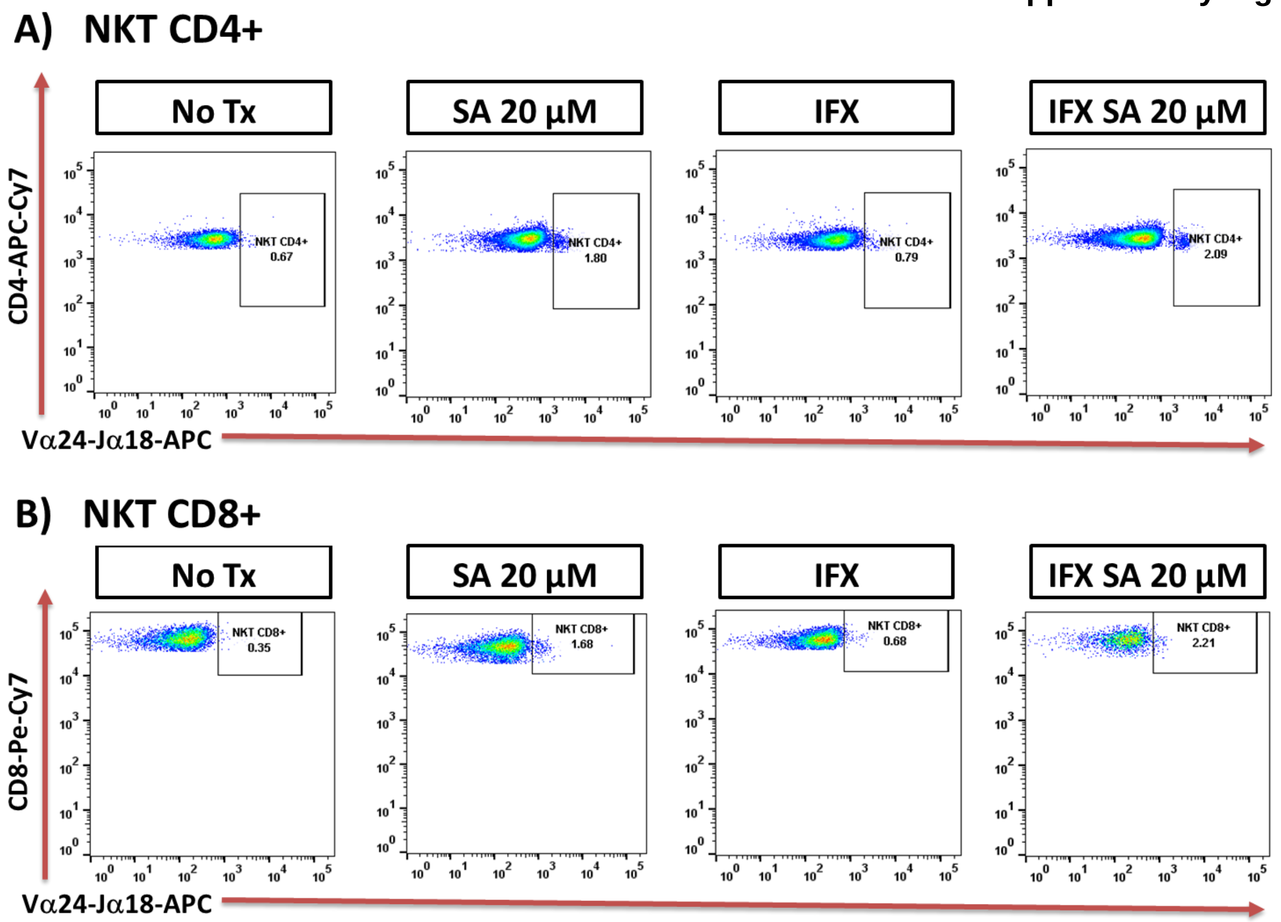

Supplementary Figure 5. Effects of simvastatin on NKTs. Representative dot plots for the effect of simvastatin on NKTs. Numbers represent the percentage of NKT CD4+ and NKT CD8+ from cells incubated under the following conditions: 20 µM simvastatin acid (SA20µM), untreated cells exposed to vehicle (0.024% DMSO and 0.056% EtOH in PBS) (No Tx), cells infected at an MOI of 0.1 (IFX), and cells infected at an MOI of 0.1 and treated with 20 µM simvastatin acid (IFX SA 20 µM).

Supplementary Figure 6

A) CD14+CD86+

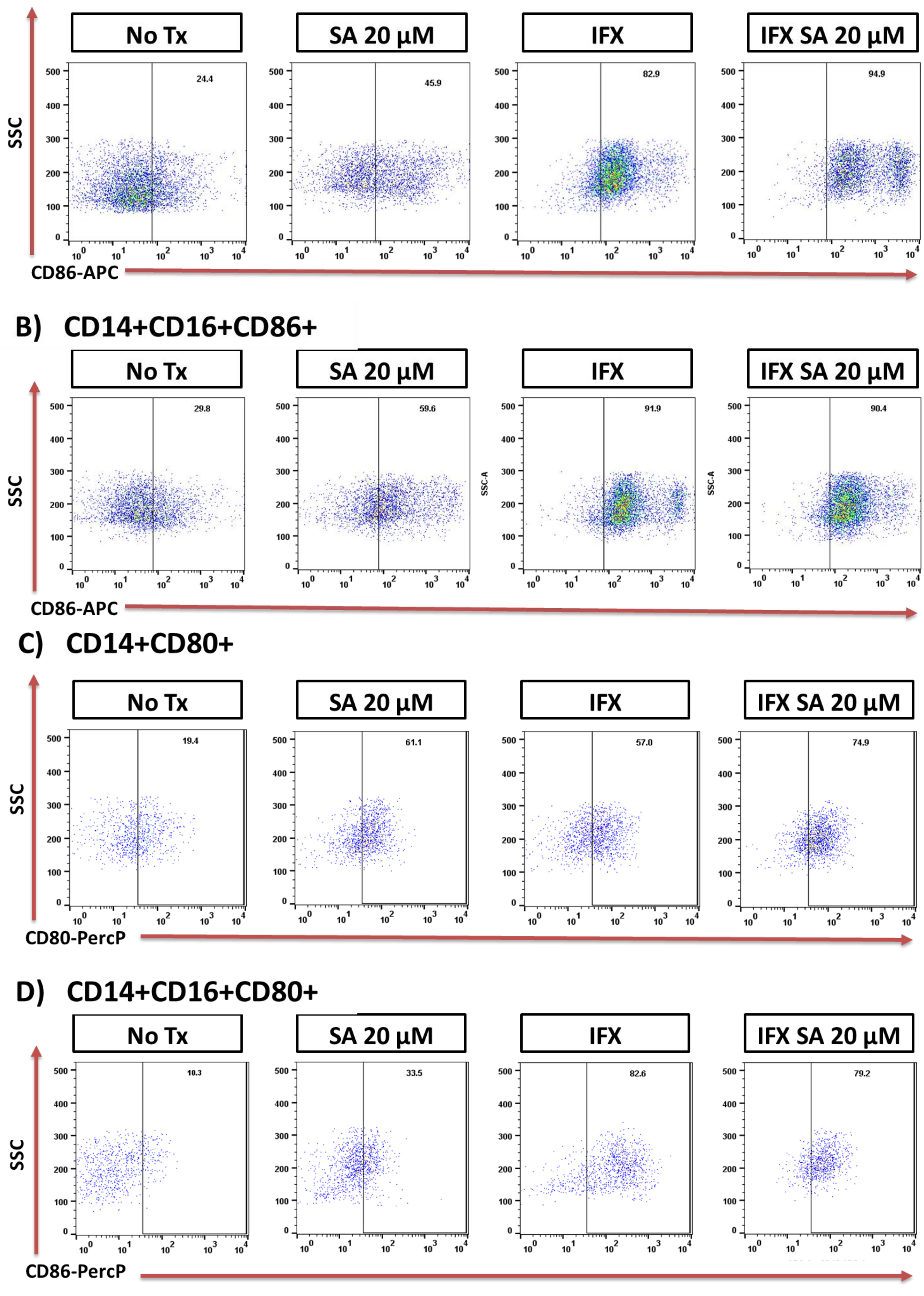

Supplementary Figure 6. Effects of simvastatin on the expression of costimulatory molecules on monocytes. Representative dot plots for the expression of CD80 and CD86 by monocytes incubated under the following conditions: 20 μM simvastatin acid (SA20μM), untreated cells exposed to vehicle (0.024% DMSO and 0.056% EtOH in PBS) (No Tx), cells infected at an MOI of 0.1 (IFX), and cells infected at an MOI of 0.1 and treated with 20 μM simvastatin acid (IFX SA 20 μM).

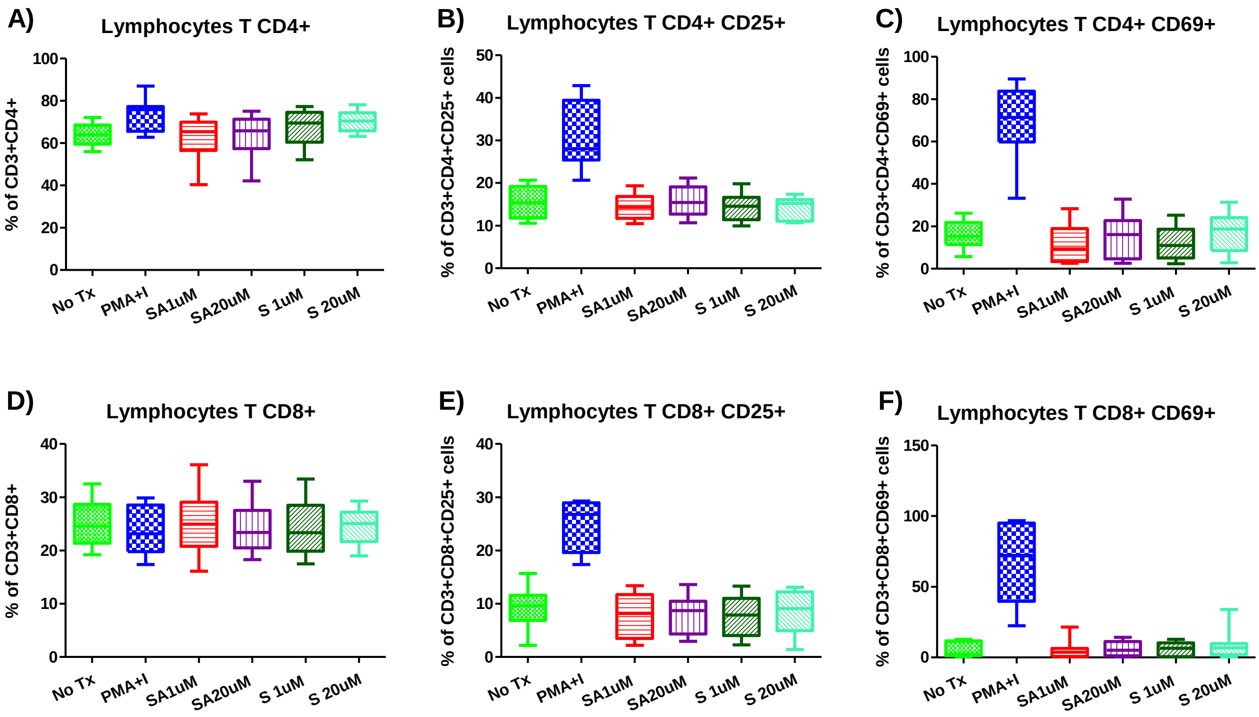

Supplementary Figure 7. Effects of simvastatin on the phenotype of uninfected lymphocytes. PBMCs were incubated in the following conditions: 1 µM simvastatin acid (SA1µM), 20 µM simvastatin acid (SA20µM), 1 µM simvastatin (S1µM), and 20 µM simvastatin (S20µM). The controls were: a) cells untreated and exposed to the vehicle (0.024% DMSO and 0.056% EtOH in PBS, NoTX), and b) cells exposed to phorbol myristate acetate (25 ng/mL) plus ionomycin (1 µg/mL) (PMA+1). After 24 hours, the phenotypes of TCD4+ (A) and TCD8+ (D) lymphocytes, as well as the activation markers CD25 (B and E) and CD69 (C and F) were evaluated, n=10. (Kruskal-Wallis, *p < 0.05 *p < 0.01).

Supplementary Figure 8

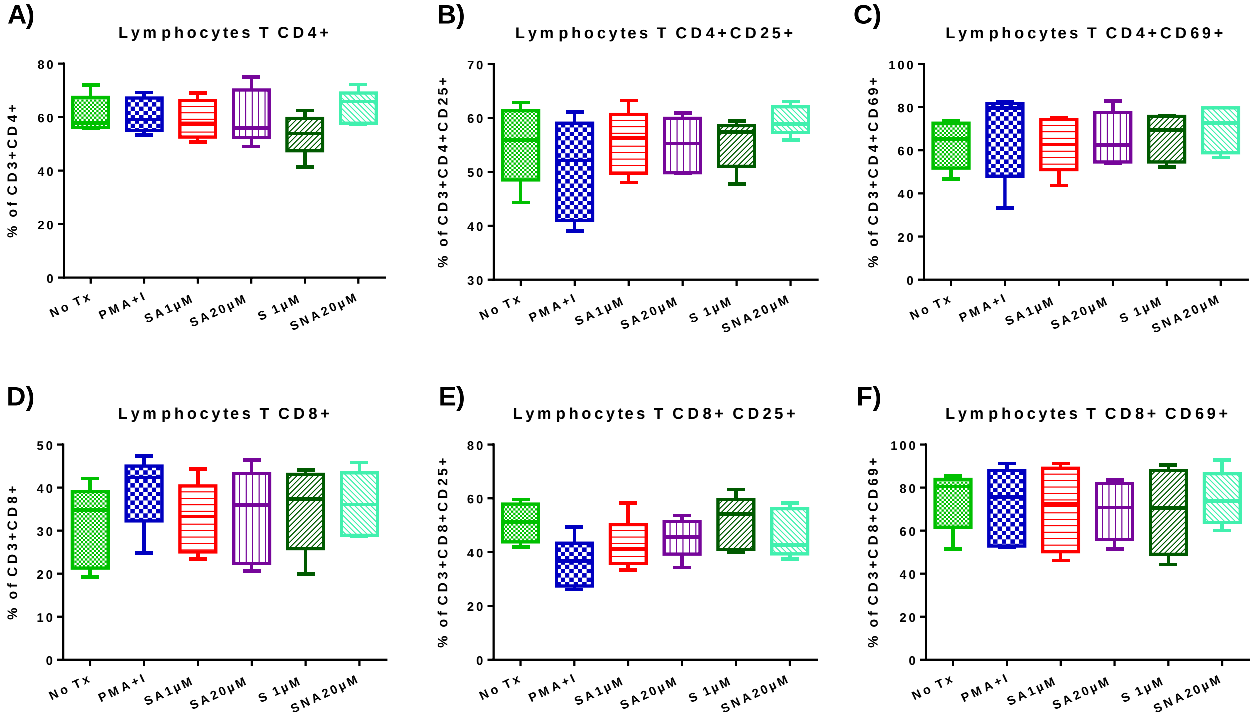

Supplementary Figure 8. Effects of simvastatin on the phenotype of lymphocytes infected with *Mycobacterium tuberculosis*. PBMCs were incubated for 24 hours in the following conditions: 1 µM simvastatin acid (SA1µM), 20 µM simvastatin acid (SA20µM), 1 µM simvastatin (S1µM), and 20 µM simvastatin (S20µM). The controls were: a) cells untreated and exposed to the vehicle (0.024% DMSO and 0.056% EtOH in PBS, No Tx), and b) cells exposed to phorbol myristate acetate (25 ng/mL) plus ionomycin (1 µg/mL) (PMA+1). Subsequently, the cells were infected with *M. tuberculosis* H37Rv at an MOI of 0.1. After 24 hours, the phenotypes of TCD4+ (A) and TCD8+ (D) lymphocytes as well as the activation markers CD25 (B and E) and CD69 (C and F) were evaluated, n=10. (Kruskal-Wallis, *p < 0.05 *p < 0.01).

Supplementary Figure 9

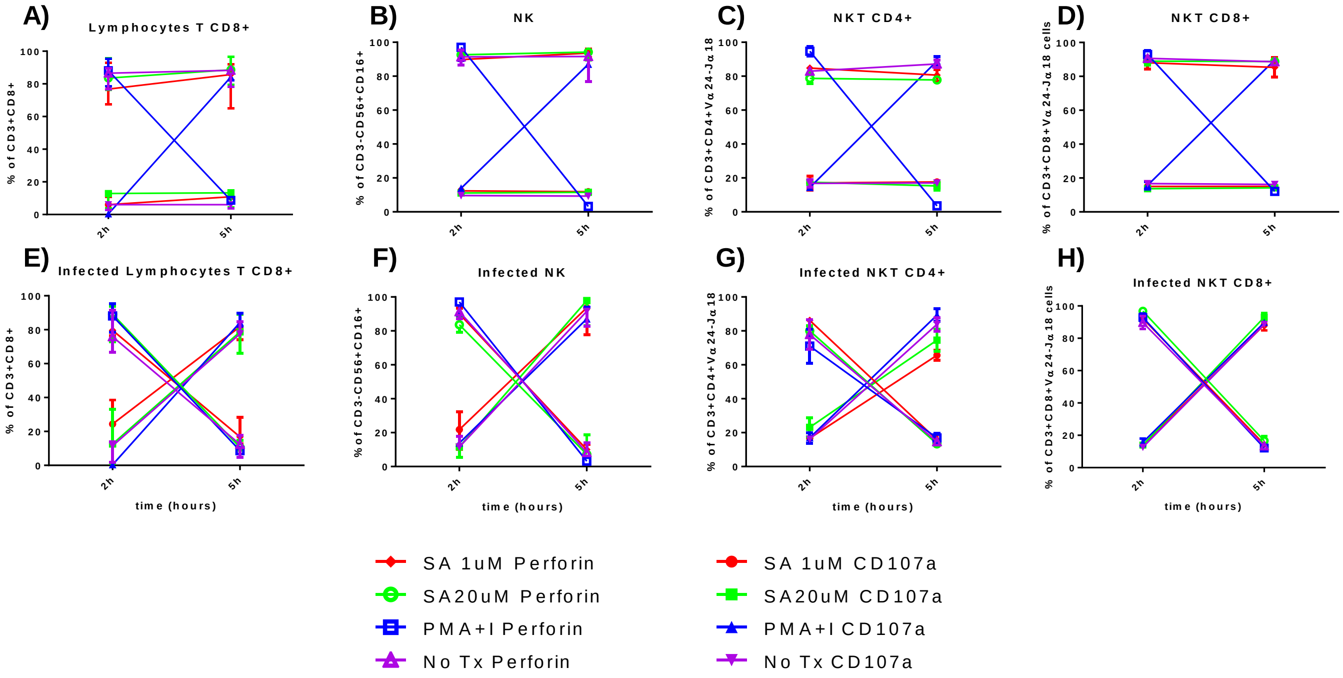

Supplementary Figure 9. Effects of simvastatin on the degranulation of cytotoxic T lymphocytes, NK cells and NKT cells. The effect of simvastatin on degranulation was evaluated in cells treated with 1 µM simvastatin acid (SA1µM), 20 µM simvastatin acid (SA20µM), 1 µM simvastatin (S1µM), and 20 µM simvastatin (S20µM). The controls were: a) cells untreated and exposed to the vehicle (0.024% DMSO and 0.056% EtOH in PBS, No Tx), and b) cells exposed to phorbol myristate acetate (25 ng/mL) plus ionomycin (1 µg/mL) (PMA+1). After 2 and 5 hours, aliquots were withdrawn, and the degranulation of TCD8+ lymphocytes (A), NK cells (B), NKT CD4+ cells (C),and NKT CD8+ cells (D) was evaluated in uninfected cells. Degranulation capacity was also evaluated in cells cultured in the same conditions and infected with *M. tuberculosis* H37Rv at an MOI of 0.1. After 2 and 5 hours post-infection, the degranulation of TCD8+ lymphocytes (E), NK cells (F), NKT CD4+ cells (G), and NKT CD8+ cells (H) was evaluated, n=10. (Kruskal-Wallis, $*p < 0.05$ $*p < 0.01$).
